# Nitrogen‐Boosted H_2_O_2_ Production of Arginine‐Polyphenol Nanozyme Drives Oxidative Eustress for Hair Regeneration

**DOI:** 10.1002/advs.202519561

**Published:** 2025-11-14

**Authors:** Yifei Wang, Yaojia Yang, Aoxue Wang, Chen Shen, Shenliang Tan, Changsheng Liu, Yuzheng Zhao, Xue Qu

**Affiliations:** ^1^ Key Laboratory for Ultrafine Materials of Ministry of Education Engineering Research Center for Biomedical Materials of Ministry of Education Frontiers Science Center for Materiobiology and Dynamic Chemistry School of Materials Science and Engineering East China University of Science and Technology Shanghai 200237 China; ^2^ Optogenetics & Synthetic Biology Interdisciplinary Research Center, State Key Laboratory of Bioreactor Engineering School of Pharmacy East China University of Science and Technology Shanghai 200237 China; ^3^ Shanghai Frontiers Science Center of Optogenetic Techniques for Cell Metabolism East China University of Science and Technology Shanghai 200237 China; ^4^ Research Unit of New Techniques for Live‐cell Metabolic Imaging Chinese Academy of Medical Sciences Beijing 100730 China

**Keywords:** hair regeneration, hydrogen peroxide, nanoenzymes, oxidative eustress, polyphenol

## Abstract

Reactive oxygen species (ROS)‐triggered oxidative eustress can stimulate regenerative signaling, yet its therapeutic window remains narrow. Mitochondrial respiratory complexes and superoxide dismutase (SOD) are canonical enzymatic sources of intracellular H_2_O_2_. Here we report a biomimetic polyphenol–amino acid nanozyme (PEAs) that couples the semiquinone radical of coenzyme Q (ubiquinone) with the Arg143 residue of Zn/Cu‐SOD1. Through self‐assembling epigallocatechin gallate (EGCG) and L‐arginine (L‐Arg), PEAs enable O_2_ adsorption and activation with controlled H_2_O_2_ generation. The H_2_O_2_ output is finely tuned by modulating the nitrogen (N) content from L‐Arg. Integrated experimental and computational analyses reveal that the N‐sites introduced by L‐Arg promote semiquinone electron delocalization, increase semiquinone abundance, thereby strengthening O_2_ adsorption, facilitating electron/proton transfer, and lowering the reaction barrier for H_2_O_2_ synthesis. Using the genetically encoded H_2_O_2_ sensor HyPerion, this work validates the sustained intracellular modulation of H_2_O_2_ by PEAs. In a mouse model of telogen effluvium, controlled H_2_O_2_ delivery activates the follicular niche via Wnt/β‐catenin upregulation and Ca^2+^/calcineurin/NFAT downregulation, resulting in robust follicle activation and a non‐pharmacological approach to alopecia therapy. This polyphenol–amino acid nanozyme therefore provides a safe and effective strategy for in vivo pro‐oxidative modulation, offering a tunable H_2_O_2_‐based platform to harness beneficial oxidative stress for tissue renewal.

## Graphical abstract

PEA nanoenzymes are assembled from L‐arginine and EGCG. The nitrogen element in arginine enhances the thermodynamic feasibility of H_2_O_2_ production through PEA's oxygen reduction catalysis, enabling nitrogen‐dependent H_2_O_2_ release. Intradermal delivery of PEA via microneedles promotes efficient H_2_O_2_ synthesis, thereby stimulating regrowth in telogen effluvium model.

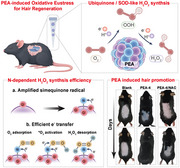

## 1 Introduction

Intracellular ROS below defined concentration thresholds participate in redox signaling and initiate physiological processes. Such species have been demonstrated to modulate cellular metabolism, augment antioxidant defense, and trigger stem‐cell differentiation, termed oxidative eustress.^[^ ^1^, ^2^, ^3^ ^]^ Among ROS, hydrogen peroxide (H_2_O_2_) is a key effector, typically eliciting oxidative eustress at concentrations of approximately 0.05–1 µM.^[^ ^4^, ^5^ ^]^ H_2_O_2_ regulates diverse signaling pathways; for example, it oxidizes the cysteine residues in nucleoprotein reductase (Nrx), inducing conformational change that prevents its binding to Dishevelled (Dvl), a mediator of the Wnt pathway, thereby upregulating Wnt/β‐catenin signaling.^[^ ^6^ ^]^ Mammalian mitochondrial ROS (mROS) influence hair follicle morphogenesis and cycling via the Wnt/β‐catenin axis. TFAMfl/fl/KRT14‐Cre^+^ mice lacking mROS display delayed hair follicle cycling due to reduced β‐catenin abundance.^[^ ^7^ ^]^ Furthermore, H_2_O_2_ redox signaling may downregulate Calcium signaling pathway.^[^ ^8^, ^9^ ^]^ It has been reported that fibroblasts undergo membrane hyperpolarization as cytosolic Ca^2+^ levels decline, which in turn stimulates hair growth.^[^ ^10^ ^]^ Harnessing controlled, H_2_O_2_‐induced mild oxidative stress to promote tissue functional renewal is therefore consonant with physiological mechanisms.

Intracellular H_2_O_2_ synthesis proceeds via a cascade of multi‐enzyme reactions: (i) redox processes catalyzed by mitochondrial respiratory complexes in concert with ubiquinone, together with NADPH oxidases (NOX), generate superoxide (O_2_·^−^);^[^ ^11^, ^12^ ^]^ (ii) O_2_·^−^ is then dismutated by superoxide dismutase (SOD) to H_2_O_2_.^[^ ^13^, ^14^, ^15^ ^]^ In this pathway, the catalytic activities of the mitochondrial complexes and SOD are dictated by key structural features: mitochondrial complex III harbors two coenzyme Q binding sites (Qo and Qi) located at the intermembrane space and within the inner mitochondrial membrane, respectively, enabling the Q‐cycle between ubiquinone and ubiquinol.^[^ ^16^ ^]^ This cycle yields ubisemiquinone, providing the principal electron donor for O_2_·^−^ formation;^[^ ^17^, ^18^ ^]^ Members of the SOD family commonly employ a conserved L‐Arg residue. In cytosolic Zn/Cu‐SOD1, the nitrogen‐bearing side chain of Arg‐143 projects toward the active‐site copper ion cavity, electrostatically attracting the anionic O_2_·^−^.^[^ ^19^ ^]^ Mutations at Arg‐143 reduce SOD catalytic activity efficiency to 1/100 of wild‐type.^[^ ^20^ ^]^ The physicochemical architecture of natural enzymes thus informs biomimetic design strategies. Integrating functional motifs from distinct enzymes at the nanoscale enables efficient, controllable catalytic systems based on H_2_O_2_‐donor nanozymes.

**Scheme 1:**
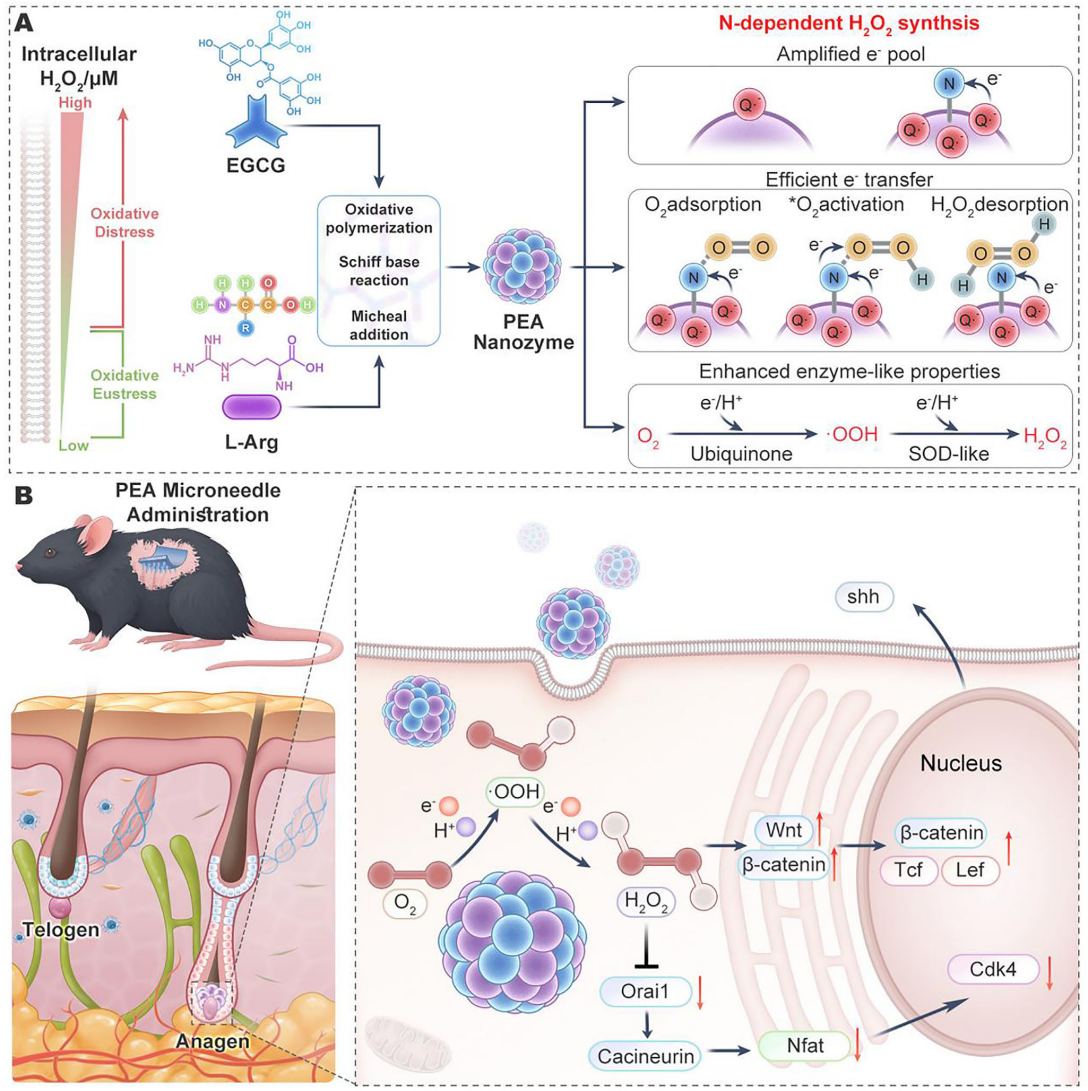
A) Construction of PEA nanozymes from EGCG and L‐arginine (L‐Arg) via oxidative polymerization, Schiff‐base condensation, and Michael addition. Nitrogen atoms from L‐Arg optimize PEA‐catalyzed oxygen reduction: first, markedly increasing the abundance of semiquinone radicals; then, providing N‐centered active sites that promote efficient O_2_ adsorption and activation, thereby enhancing the precision and efficiency of semiquinone‐mediated electron/proton transfer; and finally strengthening the ubiquinone‐like/SOD‐like cascade catalysis to release dose‐controllable H_2_O_2_ in an N‐dependent manner. B) Intradermal delivery of PEA to telogen‐phase alopecic mice using microneedles enable in situ H_2_O_2_ generation within cells of the hair‐follicle microenvironment, inducing oxidative eustress, activating the Wnt/β‐catenin pathway and down‐regulate the Calcium signaling pathways, stimulating the release of factors such as Shh and removing follicular quiescence restriction, which together accelerate the transition from telogen to anagen^[^ ^25^, ^26^ ^]^ and promote hair regeneration.

Polyphenols and amino acids are fundamental molecular building blocks in nature. The multiple phenolic hydroxyl groups of the tea polyphenol epigallocatechin gallate (EGCG) enables its function as a precursor of a semiquinone electron reservoir.^[^ ^21^, ^22^ ^]^ The essential amino acid L‐arginine (L‐Arg), bearing both a primary amine and a guanidino functionality, serves dually as a nitrogen donor and as a participant in interfacial electron transfer. In this study, we present a one‐step self‐assembly strategy for constructing polyphenol‐amino acid nanozymes (PEAs) from EGCG and L‐Arg (**Scheme** **1**A). Under alkaline conditions, the phenolic hydroxyls of EGCG are oxidized to benzoquinone, which subsequently undergoes nucleophilic attack by L‐Arg amine groups. This sequence, Schiff base formation combined with Michael addition, drives the self‐assembly process, yielding H_2_O_2_‐donor nanozymes that display both ubiquinone‐like and SOD‐like activities. Modulating the proportion of L‐Arg–derived nitrogen afforded PEAs with tunable enzyme‐mimetic performance, specifically SOD‐like activity of 1821.6–4828.3 U·mg^−1^ and an H_2_O_2_ release capacity of 0.05–0.09 µM·µg^−1^·day^−1^. Integrated experimental and computational analyses elucidate how L‐Arg nitrogen atoms influence the electron‐transfer properties of PEAs: first, lone‐pair electrons on L‐Arg nitrogen facilitate the delocalization of the semiquinone electrons and stabilize its steady‐state abundance, thereby expanding the PEA electron pool; second, L‐Arg's nitrogen sites promote a defined two‐electron transfer pathway by enhancing interfacial O_2_ adsorption, reducing the polarity of *O–O bond, and increasing local electron density at the nitrogen centers; together, these effects enable dose‐controlled release of H_2_O_2_.

The pro‐oxidative eustress efficacy of PEA nanozymes was evaluated in a telogen effluvium model (Scheme 1B). Telogen effluvium is characterized by a failure of hair follicle stem cells (HFSCs) to transition from telogen to anagen. HFSCs residing in the hair follicle bulge constitute the stem‐cell reservoir for sustained hair production. Nfatc1, a downstream effector of the Calcium signaling pathway, is highly expressed in telogen HFSCs, maintaining their quiescent state by transcribing the cell‐cycle inhibitor protein CDK4.^[^ ^23^, ^24^ ^]^ Dermal papilla cells (DPCs) enveloping the hair germ occupy the niche closest to bulge HFSCs. During the telogen ‐ anagen transition, DPCs upregulate Wnt signaling and accumulate factors such as fibroblast growth factors (FGFs), transforming growth factor‐β2 (TGFβ2), and the bone morphogenetic protein (BMP) inhibitor Noggin,^[^ ^25^, ^26^ ^]^ thereby inducing HFSCs entry into anagen. We established an in vitro HDPCs culture model coupled with the genetically encoded HyPerion H_2_O_2_ sensor,^[^ ^27^ ^]^ confirming that PEA‐4 promotes oxidative eustress in HDPCs. Intradermal administration of PEA‐4, providing dose‐controlled H_2_O_2_, to a mouse telogen effluvium model led to Wnt/β‐catenin upregulation and Ca^2+^/ calcineurin/NFAT inhibition within the hair follicle niche, accelerating HFSCs activation and hair regeneration.

## 2 Result and Discussion

### 2.1 Synthesis and Characterization of PEA Nanozymes

PEAs were constructed via a one‐step self‐assembly process involving EGCG and L‐Arg. Under mildly alkaline conditions, EGCG undergoes spontaneous autoxidation to quinones. However, the absence of amine groups yields random aggregates with poorly controlled size and morphology. By acting as cross‐linking nucleophiles, the guanidino and primary amine groups of L‐Arg participate in the oxidative polymerization of EGCG at pH 8.6, thereby facilitating the one‐step self‐assembly process. Tuning the L‐Arg/EGCG molar ratio (0–5.2) accelerated self‐assembly kinetics (Figure S1, Supporting Information) and enabled systematic control over the products’ structural and functional characteristics. The products obtained were sequentially labeled as PEGCG, PEA‐1, PEA‐2, PEA‐3, PEA‐4, and PEA‐5 (Table S1, Supporting Information).

**Figure 1:**
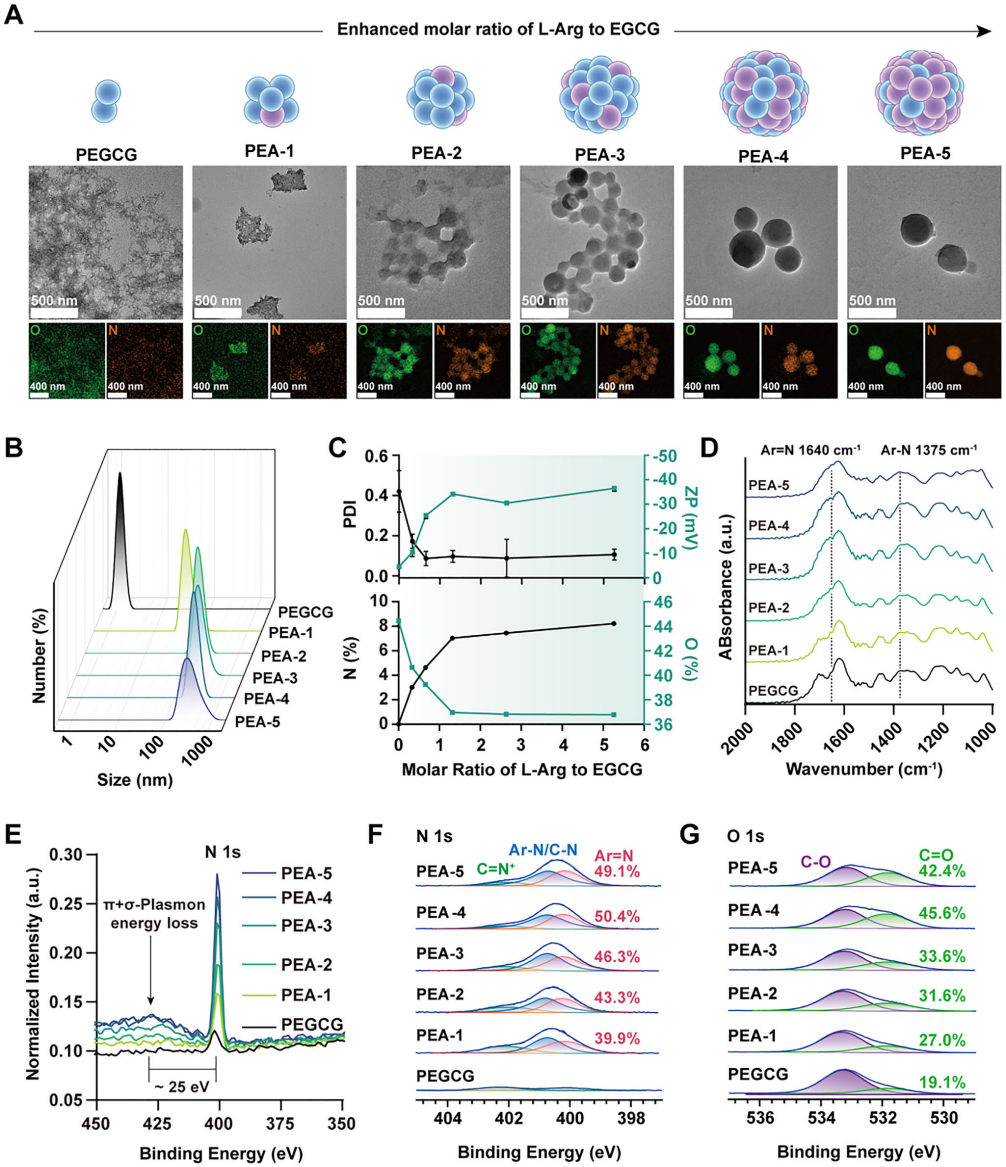
Synthesis and characterization of PEA nanozymes. A) Schematic illustration of PEA nanozymes: gradually increase the L‐Arg proportion to facilitate PEAs' formation. Below images: TEM images and EDS mappings of PEA nanozymes with different L‐Arg contents. B) DLS showing improvement in hydrodynamic size distributions. C) L‐Arg molar ratio ‐dependent PDI (n = 3), zeta potential (n = 3), N content and O content evolution. PEAs exhibit a tendency toward monodispersity and increased zeta potential with N introduction from L‐Arg. D) FTIR spectra of PEAs exhibit elevated Ar═N stretching vibration peak at 1640 cm^−1^ and Ar–N stretching vibration peak at 1375 cm^−1^. XPS of PEAs: E) survey spectrum shows enhanced N 1s abundance and energy loss peak following enhanced N content; F) high‐resolution N 1s spectra showing enhanced Ar═N content; and G) high‐resolution O 1s spectra showing enhanced C═O content.

Transmission electron microscope (TEM) analysis demonstrated a clear morphological transition from irregular flocculent agglomerates to well‐defined spherical nanoparticles, with the particle size increasing as the L‐Arg ratio increased. EDS mapping revealed progressive surface enrichment of N with significant co‐localization with oxygen (O) (**Figure** **1**A). Dynamic light scattering (DLS) showed that introduction of L‐Arg increased the hydrodynamic diameter from 5 to 440 nm. (Figure 1B). This was accompanied by a decrease in the polydispersity index (PDI) and an increase in zeta potential (Figure 1C). Collectively, these results indicate that the incorporation of L‐Arg not only promotes greater structural order during self‐assembly but also enhances the colloidal stability of the PEAs through nitrogen doping.

L‐Arg contributed to the framework construction of EGCG‐based particles, promoting the formation of regular spheres. Covalent cross‐linking mediated by highly nucleophilic amines was the primary driver. According to Fourier transform infrared (FTIR) spectroscopy, introduction of L‐Arg produced an aromatic imine (Ar═N) stretching band at 1640 cm^−1^,^[^ ^28^ ^]^ consistent with a Schiff base reaction between L‐Arg amino groups and the benzoquinone moiety EGCG. Furthermore, an Ar–N stretching band ≈1250 cm^−1^^[^ ^29^ ^]^ indicated the L‐Arg aminos also attacked the α,β‐unsaturated carbon generated upon EGCG oxidation, undergoing Michael addition at the ortho position of the quinone (Figure 1D). As a consequence of the above Schiff base reaction and Michael addition, a substantial fraction of the protonatable L‐Arg amines was consumed. Consequently, the particle‐surface zeta potential was negatively dominated by EGCG phenolic and quinone groups (Figure 1C). Consistently, analysis of the ^13^C solid‐state NMR spectrum (Figure S2, Supporting Information) showed downfield shifts of the EGCG carbons corresponding to the Schiff base reaction sites (C3', C5', C3“, C5”) and Michael addition sites (C2', C6', C2“, C6”) in EGCG after L‐Arg incorporation ,^[^ ^30^ ^]^ providing further validation for the occurrence of the two reactions mentioned above. Taken together, these observations demonstrate that L‐Arg participated in the formation of the PEA skeleton via covalent cross‐linking to oxidized EGCG units, while concomitantly extending the overall polymerization architecture.

The distribution of N and O valence states influence the formation of semiquinone, a key structural motif. Highly oxidized polyphenols favored semiquinone radical generation,^[^ ^31^ ^]^ while nitrogen incorporation was employed to regulate radical stability and electronic structure.^[^ ^32^, ^33^, ^34^ ^]^ X‐ray photoelectron spectroscopy (XPS) was used to probe electronic structures and oxidation states. The N 1s peak intensity increased with the proportion of L‐Arg (Figure 1E). Additionally, photoelectrons emitted from the PEA interior experienced energy loss via coupling to internal π and σ electrons, producing a characteristic π + σ plasmon loss feature typically 15–25 eV above the main line.^[^ ^35^ ^]^ The energy loss peak observed at 425 eV in Figure 1E correlated positively with the N 1s intensity, indicating that nitrogen introduction increased the population of unpaired/valence electrons within the PEAs.^[^ ^36^, ^37^ ^]^ Deconvolution of the N 1s spectrum showed that the Ar═N fraction rose from 39.9% (PEA‐1) to 49.1% (PEA‐5), accompanied by increased Ar–N/C–N contributions (Figure 1F), consistent with elevated cross‐link density driven by L‐Arg amines. The O 1s spectrum confirmed an increase in the C═O component from 19.1% (PEGCG) to 42.4% (PEA‐5) (Figure 1G). Enrichment of C═O in O 1s coincided with the rise in Ar═N and Ar–N in N 1s, indicating that L‐Arg accelerated EGCG oxidation to quinone and supplied a phenol/quinone pool for reliable semiquinone generation. Overall, L‐Arg not only drove assembly of size‐controllable PEA nanozymes but also significantly modulated the surface redox state, thereby yielding tunable structural and functional properties.

Compared with other amino acids, L‐Arg possessed multiple amine functionalities and an additional guanidino group, enabling assembly through covalent reactions and cation‐π interactions. Moreover, L‐Arg is a conditionally essential amino acid for humans, and its guanidino group can serve as a nitric‐oxide donor with potential to promote vascularization.^[^ ^38^, ^39^ ^]^ In conclusion, incorporation of L‐Arg rendered the PEAs tunable in morphology, chemical structure, and electronic configuration. This collective tunability established a foundation for a higher semiquinone radical population, thereby supporting efficient nanozyme‐driven H_2_O_2_ production.

### 2.2 N‐Dependent Catalytic Performance of Oxygen Reduction for H_2_O_2_ Evaluation

**Figure 2:**
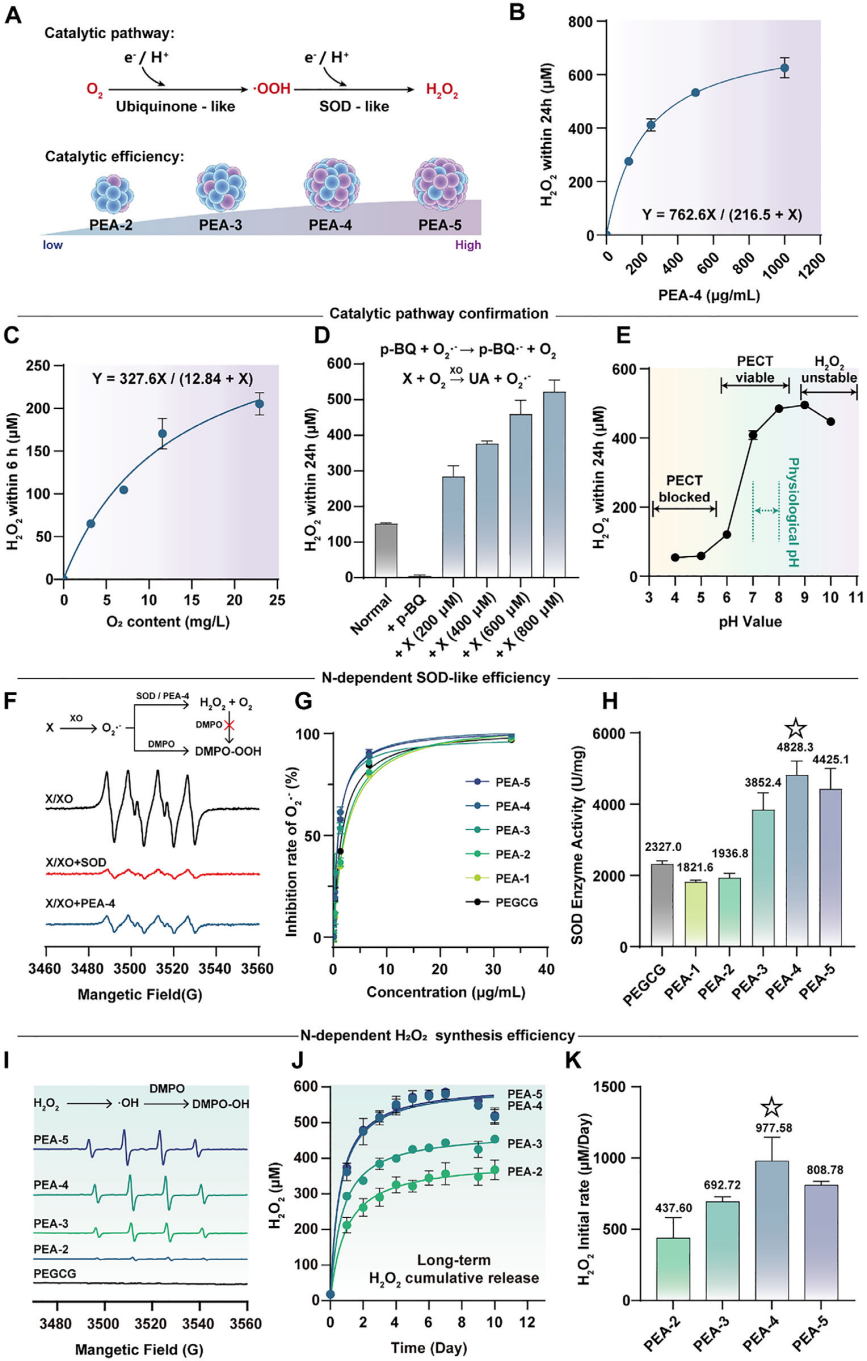
ubiquinone‐like and SOD‐like cascade catalysis conducted by PEAs. A) Schematic of H_2_O_2_ synthetic pathway and the differential synthetic efficiencies. B) Dose‐dependent H_2_O_2_ release by PEA‐4. Catalytic pathway confirmation: C) dependence of PEA‐mediated H_2_O_2_ generation on molecular O_2_; D) validation of the key intermediate in the PEA‐driven H_2_O_2_ formation pathway using the O_2_·^−^ scavenger p‐benzoquinone; E) pH dependence of H_2_O_2_ release, reflecting PCET. Nitrogen content‐dependent SOD‐like efficiency of PEA: F) EPR (electron paramagnetic resonance) verification of O_2_·^−^ scavenging; G) PEA dose‐dependent O_2_·^−^‐scavenging efficiency; H) SOD‐like activity as a function of nitrogen content, PEA‐4 exhibits the highest SOD‐like enzyme activity. Nitrogen content‐dependent H_2_O_2_ synthesis efficiency of PEA: I) EPR verification of H_2_O_2_ generation; J) H_2_O_2_‐release kinetics; K) initial H_2_O_2_‐release rates, PEA‐4 exhibits the optimal H_2_O_2_ generation. Data are shown as mean ± SD (n = 3).

EGCG is recognized as a natural antioxidant.^[^ ^40^ ^]^ However, incorporation of N from L‐Arg enabled PEAs to catalyze the stepwise, reverse reduction of O_2_ to generate H_2_O_2_: first, PEA mimicked the ubiquinone function of Complex III to initiate O_2_ reduction, producing O_2_·^−^/·OOH; then SOD‐like activity dismutated O_2_·^−^/·OOH into H_2_O_2_ (**Figure** **2**A). Quantitative analyses supported this mechanism: as shown in Figure 2B, PEA‐4 generates H_2_O_2_ in a concentration‐dependent manner, reaching a maximum cumulative H_2_O_2_ of 625 µM over 24 h and exhibiting an excellent fit to a saturation‐kinetic model (R^2^ = 0.994).

The cascade mechanism was further verified by substrate‐limitation and intermediate‐quenching experiments. Increasing the oxygen supply promoted additional H_2_O_2_ accumulation (Figure 2C), indicating that O_2_ served as the initial substrate. Moreover, using xanthine/xanthine oxidase (X/XO) as an O_2_·^−^ donor significantly amplified H_2_O_2_ production, whereas p‐benzoquinone (p‐BQ), an O_2_·^−^ scavenger, completely inhibited catalysis (Figure 2D), confirming the involvement of O_2_·^−^/·OOH intermediates. At the solid‐liquid interface, most surface redox reactions are governed by proton‐coupled electron transfer (PCET).^[^ ^41^, ^42^ ^]^ The pH dependence of PEA‐mediated H_2_O_2_ release provided indirect evidence for e^−^/H^+^ transfer in this catalytic system (Figure 2E). Under acidic conditions (pH < 5.0), excessive protonation of semiquinones impaired the e^−^‐donating capacity;^[^ ^43^ ^]^ Simultaneously, the *O–OH bond was more prone to cleavage than that the *–O bond,^[^ ^42^ ^]^ attenuating *OOH desorption and thus H_2_O_2_ generation. Under alkaline conditions (pH > 9.0), insufficient H^+^ supply^[^ ^41^ ^]^ led to decreased H_2_O_2_ stability. Ultimately, H_2_O_2_ produced by PEA peaked at physiological pH (pH 7.4), consistent with PECT's neutral‐to‐alkaline preference,^[^ ^41^, ^44^ ^]^ thereby offering a favorable therapeutic window for in vivo application. Collectively, these results indicate that PEA mediated the stepwise e^−^/H^+^ transfer to O_2_, generating H_2_O_2_ via O_2_·^−^/·OOH intermediates, in agreement with the enzyme‐cascade pathway proposed in Figure 2A.

The SOD‐like activity of PEA nanozymes is a core parameter that determines H_2_O_2_ generation capacity and exhibits N content dependency. Electron paramagnetic resonance (EPR) spectroscopy provided direct evidence for SOD‐like property (Figure 2F): PEA‐4 significantly suppressed the signal intensity of O_2_·^−^ accompanied by H_2_O_2_ generation, similar to that of natural SOD (Figure S3A, Supporting Information). The O_2_·^−^ inhibition rate of 2.5 µg mL^−1^ PEAs ranged from 80.0% to 90.5% (Figure 2G). SOD activity assays (Figure 2H) further quantified the efficiency differences among PEAs. Enzyme activity increased with the N content of L‐Arg, with PEA‐4 achieving a maximum activity of 4828.3 U mg^−1^, 2.6 times higher than that of PEAs with a lower L‐Arg ratio.

Monitoring of H_2_O_2_ synthesis further confirmed the critical role of L‐Arg‐derived N in determining the final yield. 5,5–dimethyl–1–pyrroline N–oxide (DMPO) was used to capture the downstream signals of the H_2_O_2_‐mediated Fenton reaction, enabling comparison of 24 h H_2_O_2_ output among PEAs (Figure 2I) and revealing a positive correlation between H_2_O_2_ production capacity and N content. Long‐term cumulative release profiles (Figure 2J) showed that PEA‐4 and PEA‐5 produced substantially higher cumulative H_2_O_2_ than PEA‐2 and PEA‐3, with 7‐day release tunable between 0.05 and 0.09 µM·µg^−1^·day^−1^. In addition, PEA‐4 exhibited the highest initial H_2_O_2_ release rate (0.98 µM·µg^−1^·day^−1^) (Figure 2K), consistent with the SOD activity trend in Figure 2H. Since tissue regeneration typically requires several days, PEA‐4 was selected to evaluate long‐term performance. After 14 days with daily buffer replacement, PEA‐4 maintained essentially constant daily H_2_O_2_ release (Figure S3B, Supporting Information), fluctuating between 19.5 and 17.6 µM. Collectively, PEAs struck a balance between structural stability and catalytic efficiency, thereby providing tunable and sustained H_2_O_2_ release. This capability paves the way for pro‐oxidative therapy in vivo.

In summary, L‐Arg doping conferred a ubiquinone/SOD‐mimetic cascade activity on PEAs, enabling control of H_2_O_2_ production efficiency via nitrogen content regulation—a mechanism that supports nanozyme‐mediated, benign pro‐oxidative stress therapy in vivo.

### 2.3 N‐Dependent Electrons Transfer Mechanism

**Figure 3:**
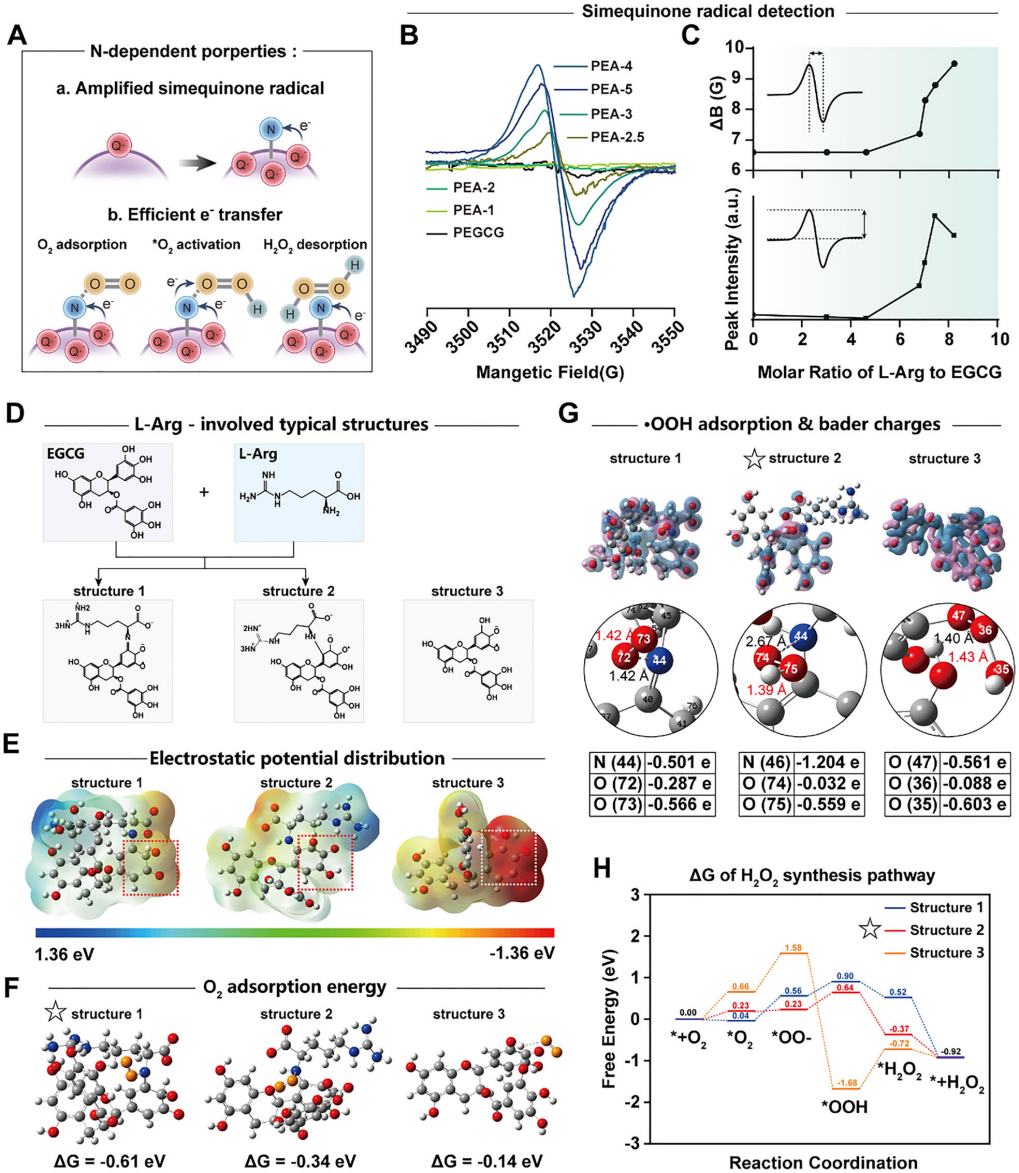
Mechanistic role of arginine nitrogen atoms in enhancing PEAs–catalyzed H_2_O_2_ generation. A) Schematic of the action mechanism of L‐arginine N atoms. B) EPR spectra showing semiquinone radical intensities for PEA with varying N contents. C) Semiquinone EPR peak line widths and intensities show positive function of nitrogen content. D) Chemical models used for DFT: structures 1 and 2 as EGCG–L‐Arg adducts formed via Schiff‐base condensation or Michael addition, and structure 3 without L‐Arg. E) Electrostatic potential maps of structures 1–3, structures 1 and 2 exhibit broader electron delocalization in the semiquinone. Red: O, blue: N, gray: C, white: H. F) O_2_ adsorption‐site configurations and corresponding adsorption energies for structures 1–3, structure 1 shows the strongest O_2_ adsorption than structures 2 and 3. Orange: O from O_2_, Red: O, blue: N, gray: C, white: H. G) Differential charge‐density distributions and Bader charges for •OOH adsorption on structures 1–3; blue and purple denote charge accumulation and depletion, respectively. Structure 2 exhibits greater N–*O bond polarity and *O–O bond stability. Red: O, blue: N, gray: C, white: H. H) Calculated reaction free‐energy profiles for the complex‐III‐like/SOD‐like cascaded H_2_O_2_ synthesis pathway on structures 1–3, the thermodynamically favorable synthesis for H_2_O_2_ is structures 2, followed by structures 1, while structures 3 is the worst.

Polyphenolic semiquinone radicals—analogous to the ubiquinone pool of mitochondrial Complex III—are valued as key electron reservoirs for driving redox cascades owing to their exceptional electron‐storage capacity. In the PEA nanozyme series, the abundant phenol/quinone moieties favored semiquinone formation, whose stability and electron‐donating capacity to substrates were further enhanced by L‐Arg N, making this modulation central to the improved H_2_O_2_ synthesis efficiency. EPR analysis (**Figure** **3**B,C) showed that L‐Arg N significantly increased the semiquinone signal and progressively broadened the spectral line widths (from 6.6 to 9.5 G), indicating higher radical density and greater spin delocalization, consistent with the gradually intensified energy‐loss peak shown in Figure 1E. Incorporation of L‐Arg N thus increased the equilibrium abundance of semiquinone radicals, thereby enabling efficient e^−^/H^+^ donation for subsequent O_2_ activation.

To elucidate the underlying mechanism, density functional theory (DFT) calculations were performed to evaluate the changes in the local chemical environment induced by L‐Arg N within PEAs. Figure 3D depicts three possible internal structures of PEAs: structure 1, the Schiff‐base condensation product of EGCG and L‐Arg; structure 2, the Michael‐addition product of EGCG and L‐Arg; and structure 3, an EGCG fragment lacking L‐Arg nitrogen. In the electrostatic potential maps (Figure 3E), electron spin near the semiquinone radical in structure 3 was more localized, increasing its reactivity toward the medium or promoting disproportionation,^[^ ^45^, ^46^ ^]^ thereby reducing semiquinone density. By contrast, in structures 1 and 2, N atoms contributed lone‐pair electrons that promoted semiquinone electron delocalization, lowering local spin density and free energy and thereby increasing semiquinone stability—consistent with the enhanced semiquinone signal observed by EPR.

Efficient H_2_O_2_ generation required a balance between O_2_ adsorption/activation and the stability of the *O–O bond.^[^ ^47^, ^48^ ^]^ The O_2_ adsorption energies of structures 1 (−0.61 eV) and 2 (−0.34 eV) were higher in magnitude than that of structure 3 (−0.14 eV) (Figure 3F), indicating that N‐centered active sites provided more appropriate adsorption strength for O_2_. Differential charge‐density maps (Figure 3G) showed electron accumulation around semiquinone O atoms and L‐Arg N atoms, whereas electron depletion localized on *OOH, indicating an electron transfer propensity from substrate to adsorbate in all three structures. In structure 1, the O (72)–O (73) bond of *OOH exhibited lower asymmetric polarity, reducing the likelihood of *O–O cleavage and thereby favoring scission of the *–O bond to release H_2_O_2_.^[^ ^49^ ^]^ In structure 2, the Bader charge at the active site N(46) was −1.204 eV, reflecting greater electron accumulation than in structure 1 (−0.501) and 3 (−0.561). Structure 2 thus provided a low‐barrier PCET channel that facilitated e^−^/H^+^ transfer to *OOH. Although the computed *O–O bond polarity in structure 2 was larger, its bond length was slightly shorter than in structures 1 and 3. Additionally, the corresponding *–O bond of structure 2 was the longest, contributing to instability and favoring *H_2_O_2_ desorption.^[^ ^50^ ^]^ By comparison, structure 3, which lacked L‐Arg N, was disadvantaged in both O_2_ adsorption/activation and stabilization of the *O–O intermediate.

DFT free‐energy calculations elucidated the barrier landscape along the catalytic O_2_‐to‐H_2_O_2_ reduction pathway (Figure 3H). Structure 2 displayed the lowest overall energy barrier, with a highest energy peak of only 0.64 eV, rendering H_2_O_2_ release thermodynamically most favorable. In contrast, the combined barriers of O_2_ adsorption/activation (+1.58 eV) and e^−^/H^+^ transfer to *OOH (+0.96 eV) in structure 3 made H_2_O_2_ release thermodynamically unfavorable. These results provide guidance for designing H_2_O_2_‐donor materials: prioritize increasing the density of Michael addition site while reducing Schiff base sites to further improve H_2_O_2_ synthesis efficiency. Overall, L‐Arg N played a dual role: it stabilized the semiquinone radical electron reservoir and constructed efficient e^−^/H^+^ transfer channels that accelerate O_2_ reduction to H_2_O_2_, collectively underpinning the amplification mechanism in PEA nanozymes.

### 2.4 In Vitro Oxidative Eustress Induced by PEA Nanozymes

**Figure 4:**
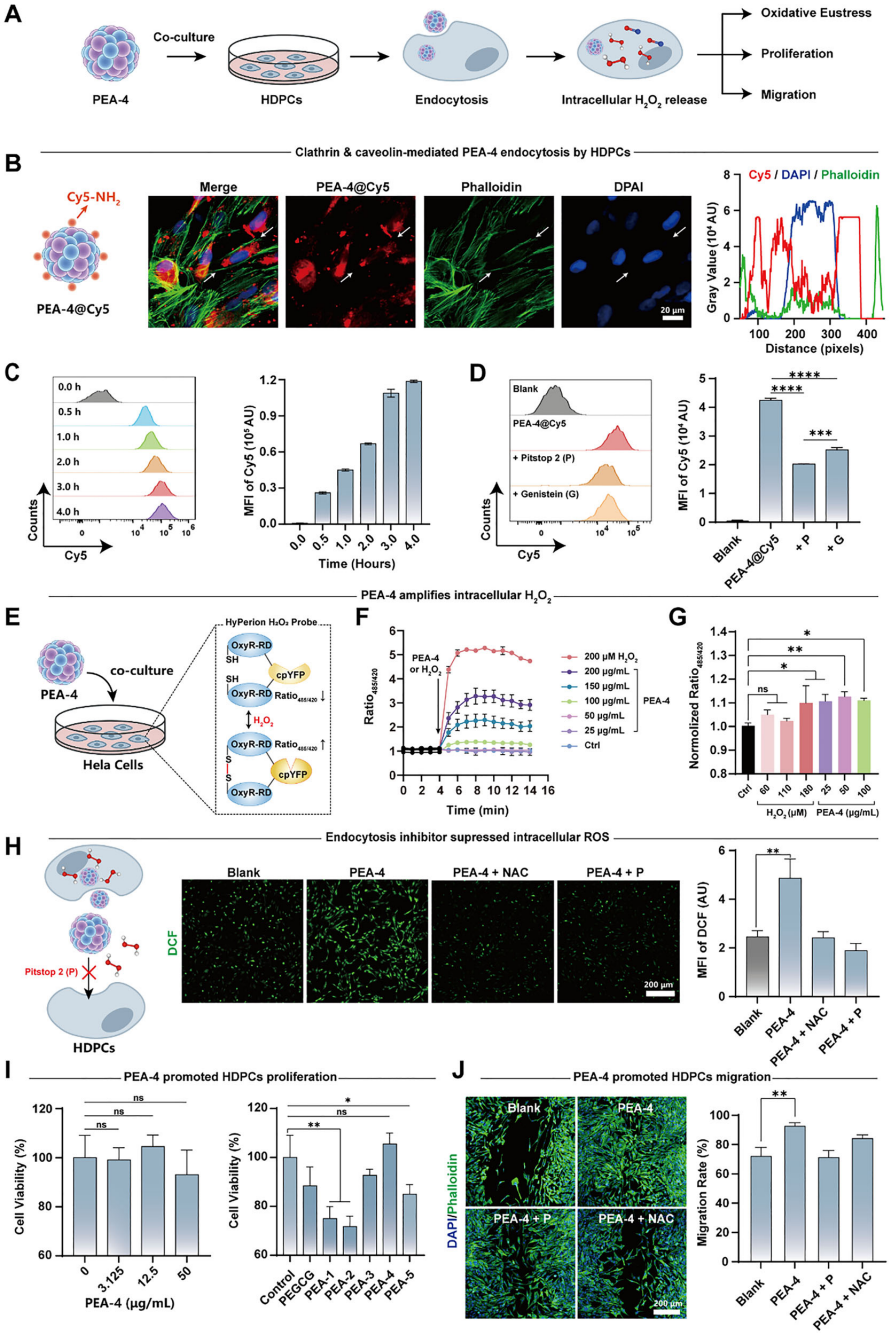
PEA nanozymes induce intracellular oxidative eustress in HDPCs. A) Workflow for verifying the oxidative eustress effects of PEA‐4 on HDPCs. Clathrin‐mediated PEA‐4 endocytosis by HDPCs: B) confocal laser scanning microscopy images showing HDPCs internalization of PEA and intracellular colocalization; C) time to equilibrium uptake of Cy5‐amine–labeled PEA particles in HDPCs quantified by flow cytometry; D) inhibition of PEA uptake by blocking caveolin and clathrin‐mediated endocytosis. PEA‐4 amplifies intracellular H_2_O_2_: E) Schematic of the procedure for intracellular H_2_O_2_ detection in HeLa cells treated with PEA‐4; F) Kinetics of the pH‐corrected HyPerion fluorescence response in HeLa cells upon different concentrations of PEA‐4 or H_2_O_2_ addition within 10 min; G) pH‐corrected fluorescence ratio detection of HeLa cells expressing HyPerion treated with different concentration of PEA‐4 or H_2_O_2_ for 24 h. Data were normalized to the fluorescence of the control. H) Pro‐oxidative modulation by PEA, endocytosis inhibitors or the reductant N‐acetylcysteine attenuate these effects. Co‐culturing with PEAs induced I) proliferation and J) 48 h migration of HDPCs, which is associated with H_2_O_2_ generated by PEAs. Data are shown as mean ± SD (n = 3) and p values were obtained by unpaired two‐tailed Student's t test. Statistical significance is set as *p < 0.05, **p < 0.01, ***p < 0.001, ****p < 0.0001; ns, not statistically significant.

The regulatory effect of tunable H_2_O_2_ release from PEA nanozymes on cellular behavior was evaluated using human dermal papilla cells (HDPCs) as the model system. DPCs constitute the mesenchymal niche at the base of hair follicles, regulating the behavior of HFSCs throughout the hair cycle. The number and signaling output of DPCs correlate directly with hair‐follicle activation.^[^ ^51^ ^]^ PEAs were co‐cultured with HDPCs, and their cellular regulatory potential was systematically assessed by quantifying PEA uptake, intracellular pro‐oxidative stress generation, and downstream effects on HDPCs proliferation and migration (**Figure** **4**A).

The investigation began with an endocytosis analysis of PEA‐4. Upon in vivo administration, parameters such as cellular uptake efficiency, tissue oxygen tension, material degradation, and metabolic clearance may limit efficient intracellular H_2_O_2_ stimulation. Therefore, PEA‐4 was selected for cellular studies owing to its efficient H_2_O_2_ release and uniform size. Cy5‐NH_2_ was used to label PEA‐4, yielding PEA‐4@Cy5 (Figure S4, Supporting Information). After co‐culturing with HPDCs, PEA‐4@Cy5 exhibited pronounced cytoplasmic accumulation (Figure 4B). Co‐localization of actin, nuclei, and PEA‐4@Cy5 confirmed successful internalization rather than surface adsorption. Flow cytometric analysis showed increasing, then saturating, mean fluorescence intensity over 4 h (Figure 4C). Pharmacological blockade with Pitstop 2 (an inhibitor of clathrin‐mediated endocytosis)^[^ ^52^, ^53^ ^]^ and Genistein (an inhibitor of clavicle‐mediated endocytosis)^[^ ^52^, ^54^ ^]^ significantly reduced cellular uptake of PEA‐4@Cy5, with Pitstop‐2 exerting the predominant inhibitory effect (Figure 4D), indicating that clathrin‐mediated endocytosis was the primary pathway for PEA‐4 uptake.

Subsequently, HyPerion/HyPerion‐c^[^ ^27^, ^55^ ^]^ probes were utilized for specific detection of intracellular H_2_O_2_ (HyPerion‐c incorporates a pH‐correction module). HyPerion was excited at 485 nm and 420 nm, emitting at 514 nm. H_2_O_2_ increased the ratio‐metric fluorescence (ratio_485/420_) (Figure 4E). Co‐culture with 100–200 µg·mL^−1^ PEA‐4 induced a concentration‐dependent rise in ratio_485/420_, which was subsequently tempered by intracellular antioxidant buffering; Consistent with the relatively slow intracellular H_2_O_2_ release kinetics of PEA‐4, direct addition of 200 µM H_2_O_2_ produced the most pronounced immediate effect (Figure 4F). After 24 h, the 25–100 µg·mL^−1^ PEA‐4 groups retained a relatively elevated ratio_485/420_; Because exogenous H_2_O_2_ was rapidly scavenged during prolonged culture, the corresponding 60 µM and 110 µM H_2_O_2_ groups showed no significant effect at 24 h, whereas 180 µM H_2_O_2_ maintained a pro‐oxidative state (Figure 4G). These results confirm that PEA‐4 achieved superior sustained intracellular pro‐oxidative modulation by delivering H_2_O_2_ in situ following cellular entry.

Furthermore, a commercial ROS probe, DCFH‐DA was used to assess the pro‐oxidative effects of PEA‐4; suppression of fluorescence by N‐acetylcysteine (NAC) verified that the signal arose from ROS. Loss of fluorescence upon Pitstop‐2 treatment further highlighted the requirement for endocytosis in sustaining intracellular pro‐oxidation (Figure 4H), consistent with Figure 4G. The SOD‐like activity of PEA‐4 was also examined in HDPCs and resulted in decreased intracellular O_2_·^−^ levels (Figure S5E, Supporting Information). In summary, limited membrane permeability and AQP3 dependence constrained intracellular exposure to extracellular H_2_O_2_. PEA‐4, with a size of approximately 350 nm and uniform spherical morphology, was well suited for clathrin‐mediated endocytosis; after internalized, it released H_2_O_2_ in situ and efficiently maintained a pro‐oxidative state.

Building on these results, we next investigated the functional cellular responses elicited by PEA‐4. Prior studies indicate that low concentrations of H_2_O_2_ function as a signaling mediator of oxidative eustress, activating MAPK/ERK, PI3K/Akt, and Wnt/β‐catenin pathways through reversible cysteine oxidation of protein phosphatases (e.g., PTP, PTEN), thereby promoting cell proliferation and migration.^[^ ^56^, ^57^ ^]^ Consistent with this paradigm, the CCK‐8 cell viability assay (Figure 4I left) revealed that PEA‐4 exhibited excellent biocompatibility with HDPCs. To directly compare the pro‐proliferative activities of PEAs differing in L‐Arg N content, we standardized the nanozyme dosage. As shown in Figure 4I (right), increasing L‐Arg N content produced an initial inhibitory effect followed by a promotional effect on cell proliferation, with PEA‐4 achieving the optimal outcome. HDPCs scratch‐wound migration assays further substantiated this profile: PEA‐4 accelerated HDPCs migration into the scratch, whereas NAC or Pitstop 2 abolished this effect (Figure 4J), indicating that the migration‐promoting activity requires intracellular H_2_O_2_ generation following PEA‐4 endocytosis.

Comparative analysis across the different PEAs revealed an intrinsic linkage between their structural attributes and biological activities. Within this series, the capacity to catalyze O_2_ into H_2_O_2_ increased with particle size, nitrogen content, and semiquinone radical density; correspondingly, cellular proliferative activity peaked at PEA‐4, implying that oxidative eustress transitions to detrimental oxidative stress once H_2_O_2_ surpasses a threshold. Additionally, the minimal cytotoxicity observed for PEGCG, PEA‐1, and PEA‐2 may be ascribed to their smaller particle sizes.^[^ ^58^ ^]^ In summary, the biological effects of PEA nanozymes are governed by their microstructure and catalytic performance, which are tuned by the L‐Arg N atom. Among them, PEA‐4 most effectively promotes oxidative eustress in HDPCs, owing to its balanced particle morphology, efficient cellular uptake, and robust H_2_O_2_‐amplifying capability.

### 2.5 In Vivo Verification of PEA‐Induced Hair Regrowth

**Figure 5:**
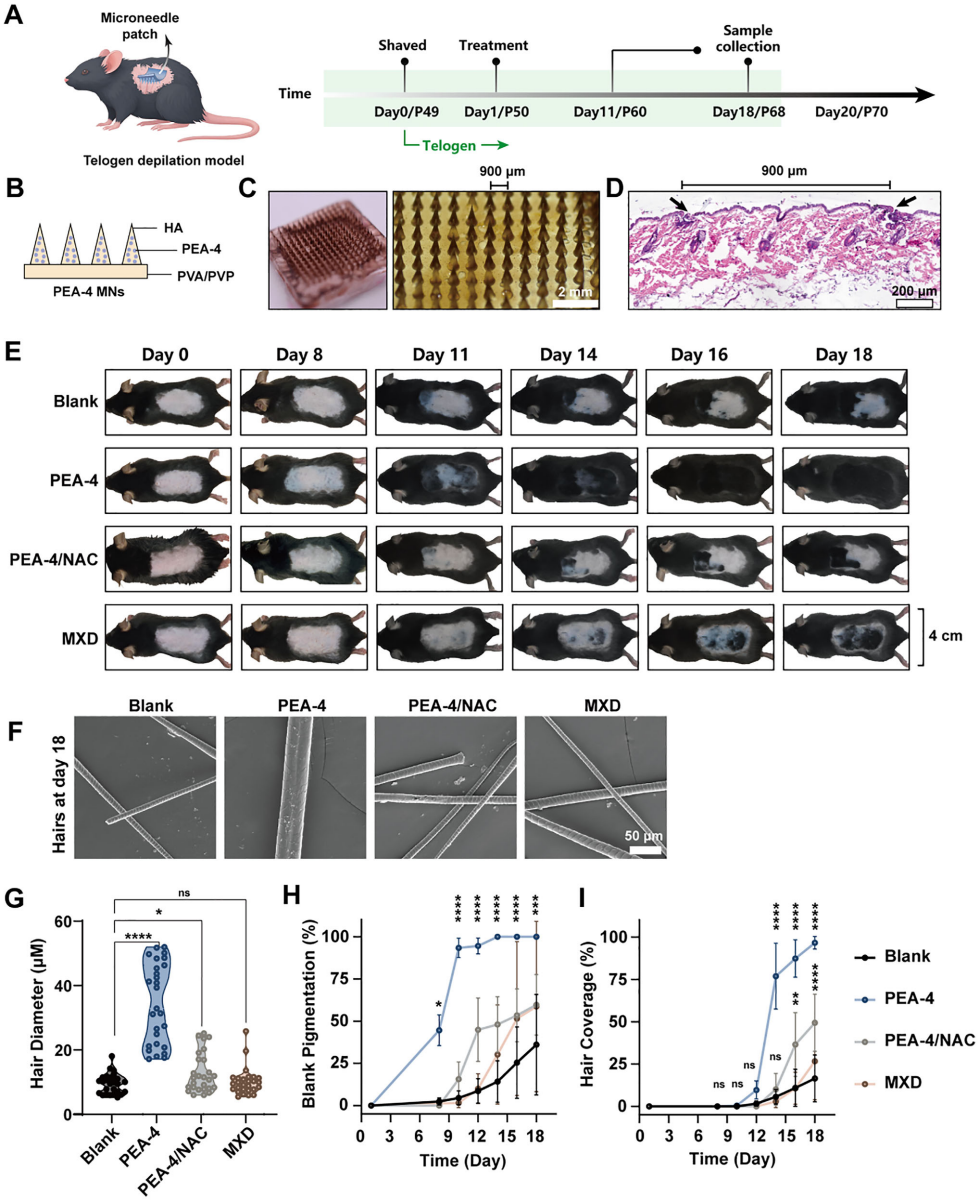
Intradermal delivery of PEA promotes hair regeneration. A) Workflow diagram for establishing the murine telogen effluvium model and in vivo delivery of PEA. B) Structural schematic of PEA‐loaded hyaluronic acid (HA) microneedles. C) Photographs of the PEA microneedle array and enlarged views of the needle tips. D) Full‐thickness mouse skin stained with hematoxylin and eosin (H&E) after application of PEA microneedles; arrows indicate two adjacent micropuncture sites created by the tips. E) Images of telogen effluvium mice at different time points. F) SEM images of mouse hair shafts. Quantitative analyses of recovery in the telogen effluvium model: G) hair shaft diameter; H) melanin‐deposited area and I) hair‐coverage area evolving curves. Data are shown as mean ± SD (n = 3) and p values were obtained by unpaired two‐tailed Student's t test. Statistical significance is set as *p < 0.05, **p < 0.01, ***p < 0.001, ****p < 0.0001; ns, not statistically significant.

To determine whether PEA nanozyme–induced oxidative eustress promotes hair regeneration, we established a telogen effluvium mouse model during the second telogen phase (postnatal day 49, P49) (**Figure** **5**A).^[^ ^59^ ^]^ We selected PEA‐4 as a representative nanozyme and administered it to the depilated dorsal skin at the second telogen (P49) using hyaluronic acid microneedles (Figure 5B). The water‐soluble microneedle patches loaded with PEA‐4 (PEA‐4 MNs) exhibited uniform, sharp tips (10–15 µm tip width), a needle height of 1550 µm (Figure S6, Supporting Information), and an inter‐tip spacing of 900 µm (Figure 5C). Hematoxylin and eosin (H&E) staining confirmed effective epidermal penetration by the PEA‐4 MNs (Figure 5D), thereby enabling targeted delivery to the follicular microenvironment within the dermis.

Daily monitoring of the skin surface over 18 consecutive days showed that mice treated with PEA‐4 exhibited the earliest onset of pigmentation and the most extensive hair coverage, surpassing the commercial minoxidil (MXD) tincture. By contrast, co‐treatment with NAC attenuated regrowth (Figure 5E). Beyond accelerated kinetics, PEA‐4 produced regenerated hairs of the highest quality—characterized by uniform, thick shafts (Figure 5F). Quantitatively, PEA‐4 significantly increased hair‐shaft diameter (Figure 5G), advanced the onset of pigmentation and augmented pigment intensity (Figure 5H), and further expanded the area of hair coverage (Figure 5I), indicating a superiority in enhancing both hair quantity and quality.

**Figure 6:**
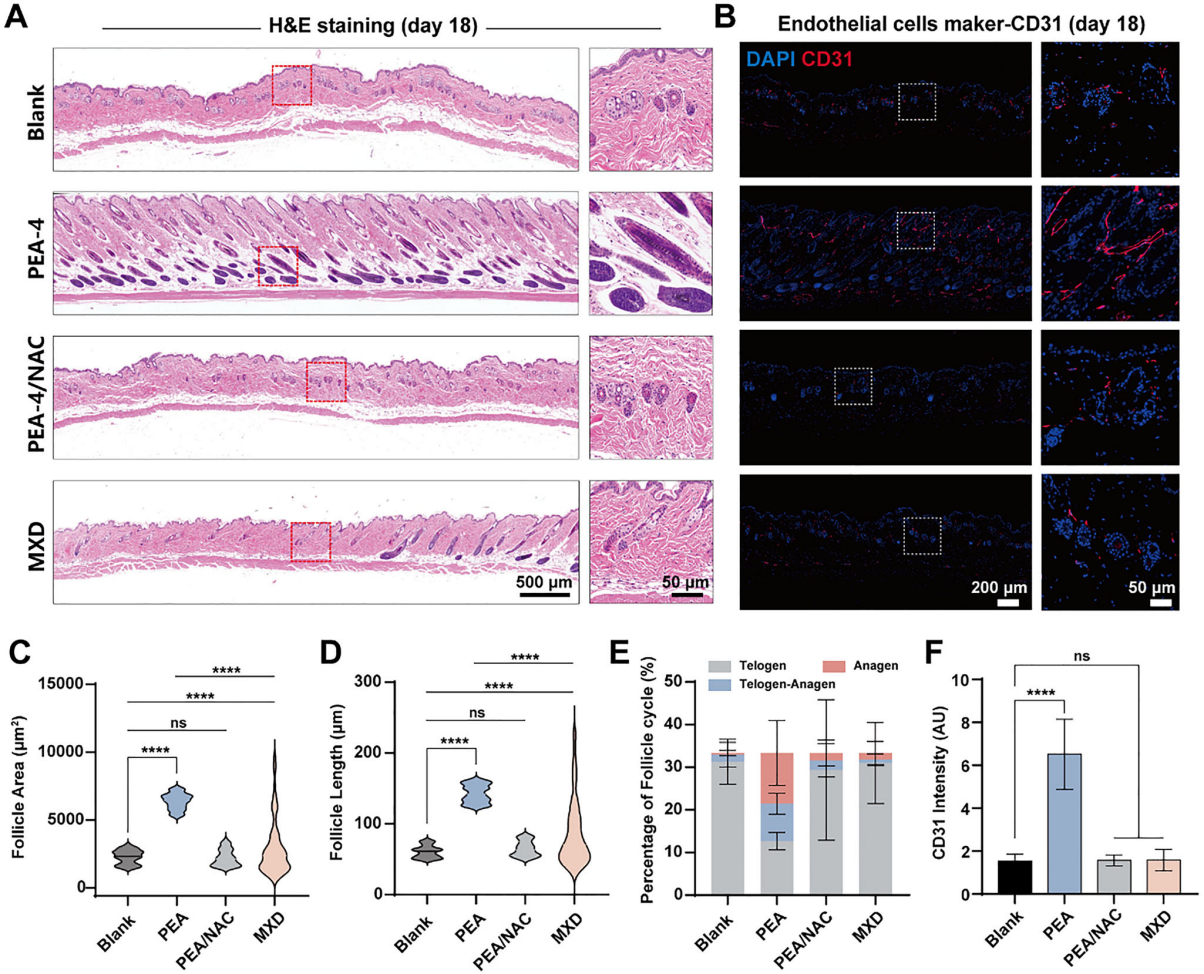
A) Day‐18 full‐thickness H&E‐stained skin sections: stitched panoramic view and enlarged images of representative hair follicles. B) Day‐18 immunofluorescence for the vascular marker CD31: panoramic view and magnified perifollicular vasculature. C–F) Quantitative analyses across groups: C) hair‐follicle area, D) hair‐follicle length, E) hair‐cycle stage distribution and F) mean CD31 fluorescence intensity. Data are shown as mean ± SD (n = 3) and p values were obtained by unpaired two‐tailed Student's t test. Statistical significance is set as *p < 0.05, **p < 0.01, ***p < 0.001, ****p < 0.0001; ns, not statistically significant.

Histological analysis revealed marked improvements in follicular morphology (**Figure** **6**A). On day 18, H&E‐stained sections from the blank group showed follicles that remained predominantly in telogen: they were superficially located, small in cross‐sectional area, and lacked prominent dermal papilla (DP) and bulb structures. In contrast, PEA‐4–treated follicles were substantially enlarged, extended deep into the reticular dermis, oriented at an angle to the skin surface, and displayed intact hair bulbs and outer root sheaths. Moreover, co‐administration of NAC nearly abolished PEA‐4′s stimulatory effect. Quantification corroborated these observations: follicle cross‐sectional area and length were maximal in the PEA‐4 group (Figure 6C,D), and follicle cycle statistics^[^ ^60^ ^]^ indicated an increased proportion of follicles in telogen‐anagen transition or anagen in the PEA‐4 group (Figure 6E).

Furthermore, hair‐follicle regeneration entailed not only intrinsic follicular activation but also a pivotal contribution from the perifollicular vasculature. Augmented neovascularization likely enhanced the delivery of nutrients and chemotactic mediators, thereby promoting coordination with follicle enlargement and hair‐cycle progression.^[^ ^61^, ^62^, ^63^ ^]^ CD31 immunofluorescence showed that PEA‐4 induced a dense microvascular plexus around follicles (Figure 6B) and yielded the highest mean CD31 intensity (Figure 6F), consistent with systemic activation of the follicular microenvironment by PEA‐4. Notably, this vascular response was abrogated by NAC, implicating a ROS‐dependent mechanism rather than nonspecific inflammation.

**Figure 7:**
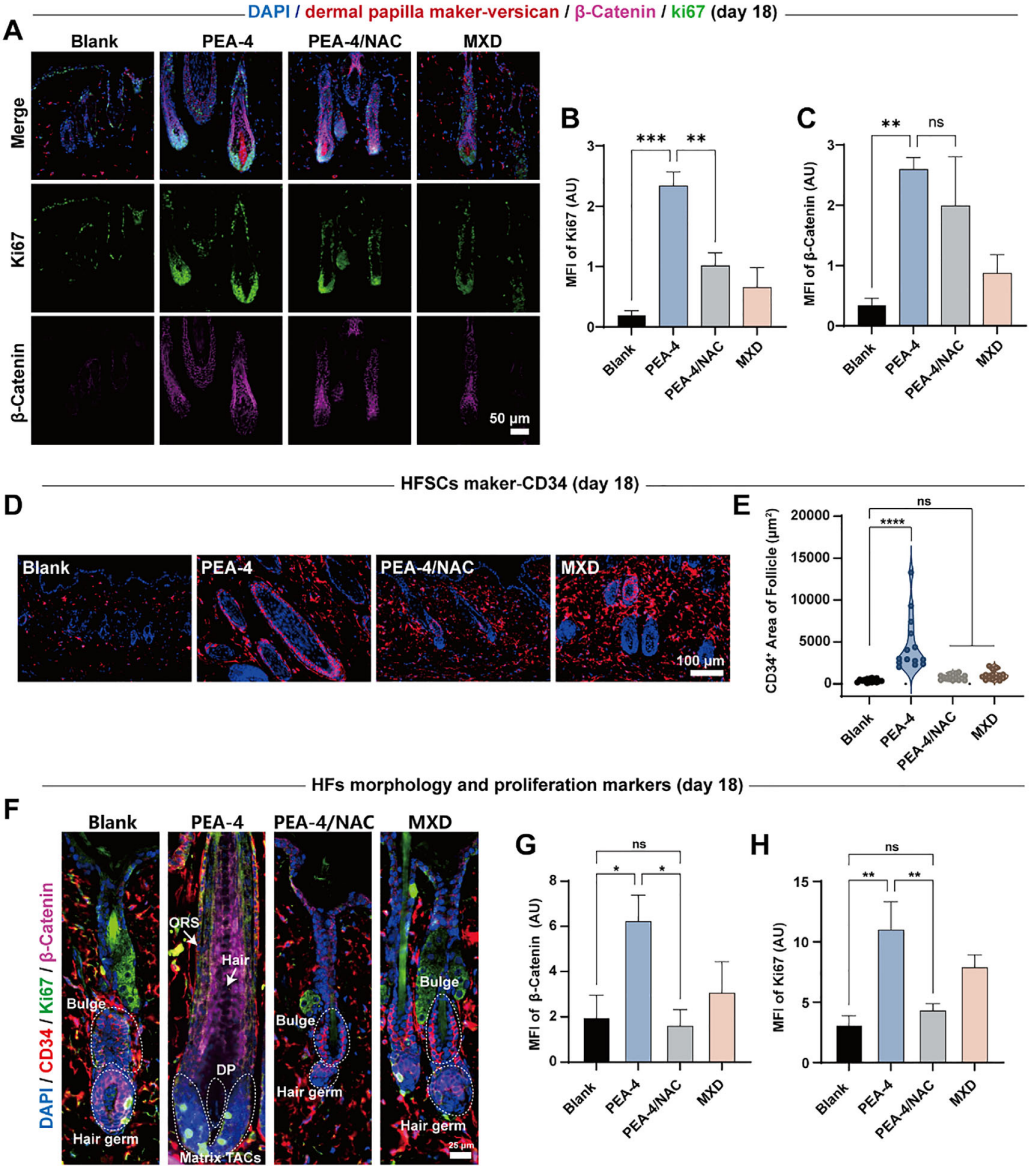
A) β‐catenin and Ki67 expression in cells adjacent to the dermal papilla within hair follicles; Versican marks the dermal papilla. Intensity quantification of B) Ki67 and C) β‐catenin. Immunofluorescence comparison of HF‐related markers at day18: D,E) HFSCs marker CD34 traces the spatial distribution changes of HFSCs, F–H) proliferation marker β‐catenin and Ki67 exhibit differences in growth signals across distinct hair follicle cycles. Data are shown as mean ± SD (n = 3) and p values were obtained by unpaired two‐tailed Student's *t* test. Statistical significance is set as *p < 0.05, **p < 0.01, ***p < 0.001, ****p < 0.0001; ns, not statistically significant.

DP is widely recognized as the key regulator that initiates hair‐cycle entry by releasing upstream mediators, including Wnt/β‐catenin, which drive quiescent HFSCs into anagen and trigger proliferation, migration, and differentiation to rebuild the follicular architecture.^[^ ^51^, ^64^ ^]^ On day 11, Versican was used to label DP specifically. PEA‐4 treatment induced substantial β‐catenin accumulation within the bulb and outer root sheath, which extensively overlapped with Ki67‐positive regions (**Figure** **7**A). The Versican signal in DP was also intensified and extended toward the bulb, indicating DP activation and transmission of anagen‐promoting cues to downstream HFSCs and transit‐amplifying cells (TACs).^[^ ^65^ ^]^ Quantification corroborated these observations: the mean fluorescence intensities of Ki67 and β‐catenin peaked in the PEA‐4 group and significantly exceeded those in the MXD group; NAC co‐treatment partially attenuated these increases (Figure 7B,C). These results demonstrate that PEA‐4‐mediated DP activation effectively engages the Wnt pathway and further suggest that a moderate amplification of ROS signaling is crucial for driving hair follicles into the anagen phase.

The observed dynamics of HFSCs provided additional support for this process. CD34 serves as a marker for HFSCs, and changes in the distribution of CD34^+^ cells reflect hair‐cycle transitions. In telogen, CD34^+^ cells are confined to a small bulge region; during the telogen–anagen transition, activated CD34^+^ HFSCs extend downward along the outer root sheath and contribute to hair‐germ formation, resulting in a significant increase in CD34^+^ area; in anagen, CD34^+^ staining becomes continuous along the sheath and reaches its maximal area.^[^ ^66^, ^67^ ^]^ On day 18, PEA‐4 ‐ treated follicles exhibited a pronounced expansion of band‐like CD34^+^ regions into the outer root sheath and hair germ, yielding the largest positive area among groups (Figure 7D,E), consistent with large‐scale HFSCs activation and progression to late anagen. In contrast, NAC co‐treatment allowed some downward migration of CD34^+^ cells, albeit with delayed kinetics compared to PEA‐4 alone. These spatial and temporal patterns align with the early DP activation described above and support a causal sequence in which the DP instructs the activation of HFSCs to initiate anagen.

With respect to signal transduction and proliferation, PEA‐4 robustly activated downstream Wnt signaling and cell‐cycle programs. PEA‐4 treated follicles displayed late‐anagen morphology: the DP was surrounded by proliferative TACs, and extensive β‐catenin and Ki67 signals were present across the bulb, inner and outer root sheaths, as well as in keratinized hair shaft regions (Figure 7F), accompanied by the highest quantitative intensities (Figure 7G,H). Follicles in control groups remained more superficial, with small bulbs and limited β‐catenin/Ki67 positivity indicative of an earlier telogen‐anagen transitional stage, lagging behind follicles treated with PEA‐4. The collective evidence established an efficient telogen‐to‐anagen transition facilitated by PEA‐4, driven by ROS‐mediated DP activation and the consequent mobilization of HFSCs.

The above‐mentioned findings indicate that the topical delivery of PEA‐4 via hyaluronic acid microneedles elicits an H_2_O_2_‐dependent activation of the follicular niche, including DP activation, HFSCs mobilization, vascular remodeling, and downstream Wnt/β‐catenin programs, which together accelerate and improve hair regeneration. The partial suppression observed with NAC indicates that controlled H_2_O_2_ signaling, rather than broad nonspecific inflammation, underlies these effects.

### 2.6 Wnt & Calcium Signalings Revealing PEA‐Induced Hair Regrowth

**Figure 8:**
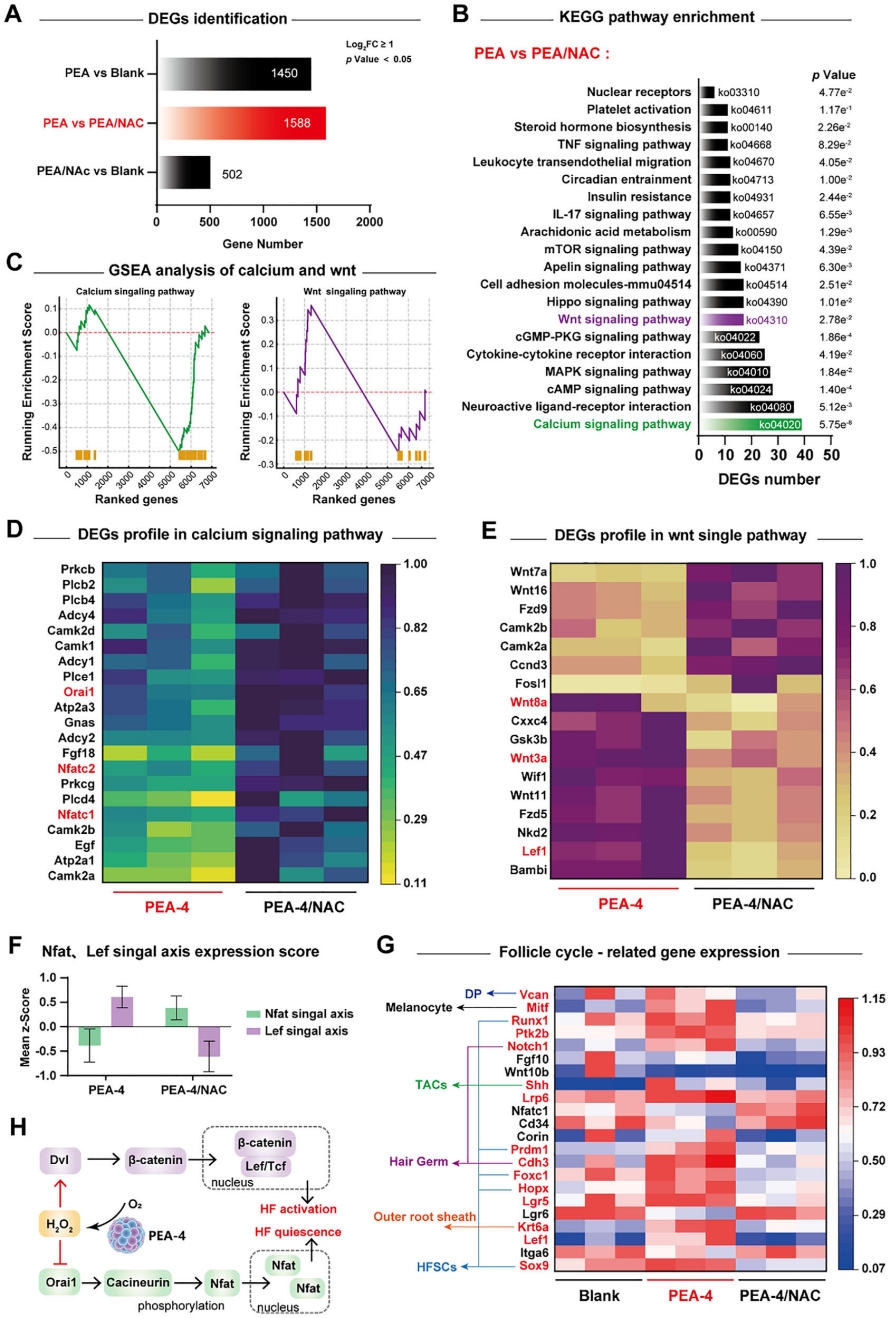
RNA‐seq analyses at day11: A) comparison of differentially expressed genes (DEGs) numbers; B) top‐20 KEGG‐enriched pathways for PEA‐4 versus PEA‐4/NAC with pathway annotations; C) GSEA of the ranked gene list shows negative enrichment of the Calcium signaling pathway and an increased trend for Wnt signaling between PEA‐4 versus PEA‐4/NAC; D) distribution of DEGs within the Calcium signaling pathway and the corresponding expression heatmap for PEA‐4 versus PEA‐4/NAC; E) distribution of DEGs within the Wnt signaling pathway and the corresponding expression heatmap for PEA‐4 versus PEA‐4/NAC; F) composite activity scores (z‐scores) for the Nfat and Lef transcriptional axes; G) heatmap of hair‐cycle–related gene expression across the Blank, PEA‐4, and PEA‐4/NAC groups with spatial‐distribution annotations. H) Operation mechanism of H_2_O_2_: H_2_O_2_ suppresses Orai1‐mediated Nfat signal and promoted Wnt/β‐catenin, thus removes HFSCs quiescent restraints and promotes proliferation. Data are shown as mean ± SD (n = 3).

RNA sequencing analysis of day 11 samples further clarified the molecular mechanisms by which PEA‐4 elicits oxidative eustress in telogen effluvium. A substantial number of differentially expressed genes (DEGs, n = 1588) were identified between the PEA‐4 group (catalytically releasing H_2_O_2_) and the PEA‐4/NAC group (in which H_2_O_2_ was scavenged by NAC), indicating that NAC remarkably reprogrammed the transcriptional program triggered by H_2_O_2_ derived from PEA‐4 (**Figure** **8**A). Kyoto encyclopedia of genes and genomes (KEGG) enrichment analysis highlighted two signaling cascades intimately associated with hair‐cycle regulation: the Calcium signaling pathway and the Wnt signaling pathway (Figure 8B). Calcium signaling maintains HFSC quiescence by elevating intracellular Ca^2+^ levels to sustain calcineurin activity, which promotes the phosphorylation and nuclear translocation of Nfat (such as Nfatc1), thereby enforcing quiescence‐associated programs.^[^ ^23^, ^24^ ^]^ In contrast, Wnt ligands (e.g., Wnt3a, Wnt8a) engage LRP5/6 to activate Dvl, leading to β‐catenin stabilization, nuclear accumulation, and cooperation with Tcf/Lef to drive proliferative gene expression, thereby promoting the transition to anagen.^[^ ^68^ ^]^ Thus, we posited that a calibrated shift in these two pathways collectively orchestrates the PEA‐4–induced telogen‐to‐anagen transition. Gene set enrichment analysis (GSEA) further revealed a negative enrichment of calcium signaling–related gene sets in PEA‐4 versus PEA‐4/NAC (p = 0.002), indicating an overall attenuation of calcium signaling that favors release from HFSC quiescence. Conversely, Wnt‐associated gene sets were positively enriched, suggesting a coordinated upregulation conducive to HFSC activation (Figure 8C). This dual‐pathway transcriptional paradigm of quiescence‐inhibition and proliferation‐activation created a favorable condition for the mobilization of HFSCs.

We next examined the DEGs enriched in the Calcium and Wnt signaling pathways. Heatmap visualization of calcium signaling components (Figure 8D) showed broad downregulation spanning from signal generation to effector execution, including Plcb2, Adcy2/Adcy4, Camk2a/2b/2d, Prkcb/Prkcg, Atp2a3, and Orai1. Downstream target evaluation indicated a lower z‐score for the NFAT signaling axis in the PEA‐4 group (Figure 8F). Suppression of the Ca^2+^–calcineurin–NFAT axis^[^ ^69^ ^]^ enforces follicular quiescence. In parallel, the DEGs heatmap of the Wnt signaling pathway (Figure 8E) demonstrated elevated expression of Wnt3a, Wnt8a, Fzd5/9, and Lef1 in the PEA‐4 group. To directly assess upstream regulators such as Dvl and β‐catenin, we calculated the z‐score of the Lef1 signaling axis, which was significantly increased in the PEA‐4 group (Figure 8F), indicating upregulation of the Dvl–β‐catenin–Lef1^[^ ^55^ ^]^ cascade associated with HFSCs activation. Moreover, co‐treatment with NAC reversed this regulatory trend in both pathways, underscoring the pivotal role of the H_2_O_2_ molecule in simultaneously suppressing the Ca^2+^/calcineurin/NFAT quiescence axis and promoting the Wnt/β‐catenin regenerative axis.

The coordinated remodeling of these dual pathways ultimately converged on the transcriptional program governing the hair follicle cycle. Heatmap analysis of follicle cycle‐related DEGs (Figure 8G) highlighted that anagen‐associated markers such as Shh, Lef1, Notch1, and Cdh3^[^ ^55^ ^]^ were systematically enriched in the PEA‐4 group but markedly diminished in the PEA‐4/NAC group, aligned with the GSEA and z‐score analyses (Figure 8C–F). Together, these data support a model in which H_2_O_2_ generated through the ubiquinone/SOD cascade of PEA‐4 establishes a state of oxidative eustress in the follicular microenvironment: on the one hand repressing calcium‐dependent HFSC quiescence, and on the other stabilizing Dvl and enhancing β‐catenin/Lef transcriptional output. These combined effects synergistically propelled the follicular cycle from telogen toward anagen (Figure 8H).

It is noteworthy that the PEA‐4‐induced dual modulation of Calcium and Wnt signaling not only clarifies the molecular basis for its promotion of hair follicle regeneration but also points to broader translational applications. For instance, in ischemic stroke, acute‐phase calcium overload should be curtailed to prevent excitotoxicity, followed during the subacute and chronic phases by selective activation of Wnt/β‐catenin signaling to promote neural stem/progenitor cell proliferation, differentiation, and angiogenesis.^[^ ^70^, ^71^, ^72^, ^73^ ^]^ Similarly, in cardiovascular disease, inhibition of calcium signaling reduces vascular tone and ameliorates myocardial ischemia, whereas transient, spatially confined activation of Wnt facilitates tissue repair.^[^ ^74^ ^]^ In models of immune tolerance and transplantation, activation of Wnt may facilitate the establishment of local tolerance, while concomitant inhibition of the Calcium/calcineurin/NFAT pathway can mitigate acute immune responses.^[^ ^75^, ^76^ ^]^ The tunable H_2_O_2_ release profile of PEAs enables their precise adaptation to these conditions, highlighting their versatility for diverse applications.

## 3 Conclusion

In summary, this study presents the construction of nanozymes (PEAs) from natural tea polyphenol EGCG and the essential amino acid L‐Arg. The biomimetic integration of key ubiquinone‐ and SOD‐derived motifs at the nanoscale affords tunable H_2_O_2_ production, constraining release within the concentration window that elicits oxidative eustress. Mechanistically, this N‐dependent regulation of H_2_O_2_ synthesis arises from its effects on semiquinone abundance, interfacial O_2_ adsorption/activation, and H_2_O_2_ desorption. Moreover, the mode of nitrogen incorporation critically determines catalytic efficiency: N introduced via Michael addition favors the thermodynamics of H_2_O_2_ generation relative to Schiff‐base condensation.

PEA‐4 was identified as an effective modulator of intracellular pro‐oxidative signaling. In vivo, PEA‐4 upregulated Wnt/β‐catenin signaling and downregulated the Ca^2+^/calcineurin/NFAT pathway, thereby orchestrating dual‐pathway regulation that accelerated the transition of HFSCs from telogen to anagen. Beyond this application, the metal‐free, non‐immunogenic, and drug‐free nature of programmable pro‐oxidative PEA nanozymes provides a generalizable platform to achieve tissue rejuvenation in other contexts governed by ROS dose dependence.

## 4 Experimental Section

### Materials

L‐Arginine (98.5–101.0%), o‐phthalaldehyde (OPA, ≥99%) was purchased from Sigma‐Aldrich Co. Epigallocatechin gallate (99%) was obtained from Bide Pharmatech Ltd. DL‐Dithiothreitol (DTT, >99%), 2′,7′–dichlorofluorescin diacetate (DCFH‐DA), cell counting kit‐8 (CCK‐8), dihydroethidium (DHE) and 3–amino, 4–aminomethyl–2′,7′–difluorescein, diacetate (DAF‐FM diacetate) were obtained from Shanghai Beyotime Biotech Co., Ltd. Hydrogen peroxide (H_2_O_2_) was acquired from Sinopharm Chemical Reagent Co., Ltd. Sodium dodecyl sulfate (SDS, 99%) was obtained from Meryer (Shanghai) Chemical Technology Co., Ltd. Hyaluronic acid (HA, Mw = 10 kDa, 99%) was purchased from Shanghai Yien Chemical Technology Co., Ltd. HA (Mw = 800–1000 kDa) was obtained from Heowns Biochem LLC. Minoxidil (5%) was purchased from Wansheng Pharmaceutical Co., Ltd. SOD Assay Kit was obtained from Dojindo Laboratories. P‐benzoquinone (p‐BQ, ≥99.5%), polyvinyl alcohol (PVA, Mw≈145 kDa) was purchased from Aladdin. Polyvinylpyrrolidone (PVP, K90) was obtained from Shanghai Xushuo Biotechnology Co., Ltd. Amplite fluorimetric hydrogen peroxide assay kit was sourced from AAT Bioquest. Cyanine5 amine (Cy5–NH_2_), xanthine (X, 98%) and xanthine oxidase (XO, 50 U × mg^−1^ protein) was purchased from Shanghai Yuanye Bio‐Technology Co., Ltd. All cell culture related reagents were obtained from Gibco.

### Synthesis of PEA Nanoparticles

Initially, different concentrations of L‐arginine solution were prepared. Then 1000 mg of EGCG was completely solubilized in 180 mL deionized water. Once the solution was stable at 26 °C with intense stirring at 400 rpm, 20 mL of L‐Arginine solution was added to the reaction system and the pH value was rapidly adjusted to 8.7 using NaOH (1 M). PEA NPs were collected and purified through ultrafiltration (30 kDa, 4500 rpm) or centrifugation after the different reaction times. All of the above nanoparticles were finally freeze dried and stored at −20 °C.

### Synthesis of PEA@Cy5 Nanoparticles

PEA (1 mg × mL^−1^) was resuspended in PBS (pH 7.4, 0.1 M), then the pH value was adjusted to 8.6 using 1 M NaOH. After the addition of Cy5‐NH_2_ (320 µL, 40 µg × mL^−1^), the mixture was allowed to react for 24 h. PEA@Cy5 nanoparticles were obtained by centrifugation (12 000 rpm, 15 min) and washed with deionized water for several times.

### Characterization

Morphology images of PEA nanoparticles were obtained by field emission transmission electron microscopy (Talos F200X). Dynamic laser light scattering spectrometer (ALV/CGS‐5022F) was employed to measure the size and zeta potential of different samples. A multi‐function enzyme labeler (SpectraMax M3, USA), elemental analyzer (vario EL cube) and X‐ray photoelectron spectrometer (ESCALAB 250Xi) were used to perform chemical analysis of the samples. Electron paramagnetic resonance (Bruker EMX‐8/2.7) was utilized to detect semi‐quinone radicals of PEA. FTIR spectra were recorded by an FTIR spectrometer (Thermo Nicolet 6700). Ultraviolet absorption spectrum was recorded by the ultraviolet‐visible spectrophotometer (PerkinElmer Lambda 950).

### Electrochemical Reverse Engineering

PEA (0.3 mg) nanoparticles are air‐dried onto the 1 × 1 cm^2^ carbon paper electrodes (Toray 060) respectively. Then they were tested by the cyclic voltammograms (CVs) in the presence of soluble mediators (1 mM Fc in 0.1 M PB, pH 7.4). Scan rate 10 mV/s.

### Evaluation of SOD Enzyme Mimicking Activity

Following the manufacturer’ s instructions (Dojindo), the SOD‐mimicking activity of PEA NPs was assessed. The absorbance of the resulting product, water‐soluble tetrazolium salt‐1 (WST‐1) formazan, was measured at 450 nm employing a multi‐function enzyme labeler. Kinetic curves and SOD‐like activity were calculated from the inhibition percentage of the WST‐1 reaction with O_2_·^−^.

### In Vitro L‐Arginine Release

In vitro L‐arginine release profile of PEA NPs were performed using PBS (pH = 7.4 containing 1% SDS or pH = 6.0 containing 1% SDS) under 37 °C. The fluorescent productions derived from OPA was used to quantify the release behavior of L‐arginine. Specifically, OPA was dissolved in a mixture of Na_2_B_4_O_7_·NaOH buffer solution (0.2 M, pH 9.8) and methanol, followed by the addition of DTT. Subsequently, the sample solution (15 µL) was mixed with above mentioned derivatization reagent (150 µL) for 3 min. The fluorescence intensity (λ_ex_ = 340 nm, λ_em_ = 450 nm) was recorded using a multi‐function enzyme labeler. The cumulative release amount of L‐arginine was calculated as:

(1) $$\mathrm{Cumulative}\;\mathrm{Release}\;\mathrm{Amount}=V\times Q_{t}+vQ_{t-1}+vQ_{t-2}+\cdot \cdot \cdot +vQ_{0}$$

In the equation, *V* is the total volume of the release system, *v* is the volume of sample solution extracted. *Q_t_* is the L‐arginine concentration in the sample solution at time *t*, *Q_0_* is the initial release amount.

### Detection of H_2_O_2_ Generation

PEA NPs was dispersed in buffers and shaken in 37 °C for H_2_O_2_ release. After incubation for different times, H_2_O_2_ generation was measured by Amplite Fluorimetric Hydrogen Peroxide Assay Kit.

### Detection of ·OH

Electron paramagnetic resonance spectroscopy (EPR) assay was carried out on a Bruker EMX‐8/2.7 spectrometer at room temperature. PEA nanoparticles were incubated with PBS (pH 7.4, 0.1 M) for 4 days; then the supernatant was mixed with FeCl_2_ (1 mM) and DMPO (100 mM) and examined rapidly.

### Detection of O_2_·^−^

To examine the ability of PEA to scavenge O_2_·^−^, xanthine (X)/ xanthine oxidase (XO) system was used to generate O_2_·^−^. After adding different concentrations of PEA‐4 to the system, the reaction was initiated by the introduction of XO. 100 mM DMPO was used to trap O_2_·^−^. The EPR measurements were carried out using a Bruker EMX‐8/2.7 spectrometer at ambient temperature.

### Theoretical calculations

Density functional theory (DFT) calculations were carried out using Gaussian 09 programs^[^ ^77^ ^]^ throughout this manuscript. Geometric optimizations were performed using B3LYP hybrid functional with 6‐31G(d) basis set. The Grimme's DFT‐D3 method was applied to correct the van der Waals for all molecules. Vibrational frequency analyses were performed using B3LYP hybrid functional with 6‐311G (d, p) basis set for all stationary points to verify whether each optimized structure corresponded to a local minimum. All minima, including reactants, products, and intermediates, displayed no imaginary frequencies. The free energies (G) of various intermediates are defined as ΔG = Ei − Ereactant, where Ei is the energy of intermediates or transition state structures, and Ereactant is the total energy of reactants.

When the optimization was completed, the binding energies (ΔE) of O_2_ with 1, 2, and 3 were calculated as

(2) $$\Delta E\left(eV\right)=27.212\times \left(E_{total}\left(Ha\right)-E_{1}\left(H_{a}\right)-E_{2}\left(Ha\right)\right)$$

where the E_total_ is the energy of the optimized system; E_1_ is the energy of the 1, 2, and 3; E_2_ is the energy of O_2_.

### Fabrication of PEA Loaded Dissolving Microneedles

PDMS molds with conical cavities (*D* = 580 µm, *h* = 1550 µm) array (15 × 15, *d_cc_* = 900 µm) were used to fabricate microneedles. PEA NPs were mixed with 10 wt% HA‐10 kDa and 10 wt% HA‐800 kDa. After deposited into the needle cavities by centrifugation, the excess liquid was removed for base patch cast containing 15 wt% PVA and 5 wt% PVP. Finally, the patches were dried at 37 °C for 12 h before detached.

### In Vitro Biocompatibility Assessment

Human dermal papilla cells (HDPCs) were cultured in DMEM medium supplemented with 10% fetal bovine serum in a 5% CO_2_, humidified atmosphere at 37 °C.

To assess the cytotoxicity of nanoparticles, HDPCs were seeded in 96‐well plates with a density of 3.0 × 10^3^ cells per well. After 24 h incubation, the fresh medium containing PEA NPs (50 µg × mL^−1^) were added and incubated for another 24 h. Furthermore, different concentrations of PEA‐4 were also performed the same steps as above. Untreated group was taken as a negative control. Then cells were incubated for 24 h at 37 °C under 5% CO_2_ and the cytotoxicity was evaluated by the CCK‐8 assay. The fluorescence images of cell proliferation every 24 h were acquired by laser confocal microscope (Nikon, Japan).

### In Vitro Migration Assay

HDPCs were inoculated in the medium and cultured in 5% CO_2_ at 37 °C. After 24 h, the cell monolayer (≈320 µM) was scratched using a 200 µL pipette tip. Different concentrations of PEA‐4 were added to the culture medium (containing 0.5% fetal bovine serum). The images were taken with an inverted phase contrast microscope at 0, 24, 36, and 48 h.

### Cell Endocytosis Effect of PEA

HDPCs and PEA@Cy5 were employed to investigate the cell internalization of PEA. The HDPCs were incubated with PEA@Cy5 for 4 h. After incubation, the cell nucleus and skeleton were co‐stained with DAPI and Rhodamine Phalloidin. Then the confocal fluorescence images were recorded. A flow cytometer (Beckman coulter, USA) was also used to quantify the intracellular fluorescence of Cy5 after trypsin digestion.

### In Vitro ROS Generation

HDPCs were employed to study the ROS generation ability of PEA in vitro. DCFH‐DA probe was utilized to detect the intracellular ROS.

First, HDPCs were seeded on confocal dishes for 24 h. Then, the cells were pretreated with EGCG, L‐Arg and PEA‐4 for 6 h. Additionally, vitamin C was introduced to remove the H_2_O_2_ produced by PEA as a control. Finally, 5 µM DCFH‐DA was added and allowed to incubate for 30 min at 37 °C. Besides, HDPCs were pretreated with PEA‐4 for 4 h. Then DHE was served as a peroxide indicator.

### Assessment of Intracellular Redox State

To generate the stable cell lines, Hela cells were transfected with the pLVX lentiviral plasmids encoding one sensor, produced by co‐transfecting two lentiviral packaging vectors (pMD2.G and psPAX2) and plasmid of one sensor in HEK293T cells. Hela cell lines were seeded into 96‐well plates with a density of 12 000 cells per well for 12 h. Then cells were cultured with PEA‐4 or H_2_O_2_ PEA‐4 (25, 50, 100 µg mL^−1^) or H_2_O_2_ (60, 110, 180 µM) for 24 h. Subsequently, cells were washed twice with HBSS with 100 mM HEPES and 25 mM glucose (pH 7.4) at 37 °C after removing the medium. Data were recorded immediately after the addition of 100 µL HBSS with 25 mM glucose at 37 °C by a Synergy NeO_2_ Multi‐Mode Microplate Reader (BioTek) with 420 BP 27 nm and 485 BP 20 nm excitation filters and a 532 BP 40 nm emission filter. Fluorescence values were background corrected by subtracting the intensity of the cell samples not expressing sensors.

### In Vivo Assessment of Hair Growth

The hair regeneration capabilities of the microneedles were evaluated in male C57BL/6 mice (6 weeks old, Beijing leagene biotech. Co, Ltd). All animal experiments were performed in compliance with the Animal Research Committee of the Institutional Animal Care and Use Committee of SHZY (SHZY‐202108211). For in vivo therapy experiment, animals were acclimatized to the new environment for 1 week and were shaved at postnatal day 49. The mice were randomly divided into four groups: Blank‐MNs, PEA4‐MNs, PEA4‐MNs/NAC and 5% minoxidil. Mice were depilated on day 0 and the microneedles and minoxidil were applied on day 1, minoxidil was administered topically at a dosage of 100 µL cm^−2^. Digital pictures of skin were taken. The time profile of the hair phenotype transformation was derived from real‐time observation of the hair regrowth in mice.

### Skin Tissue Histology

The skin on the back of the mice was taken for H&E staining and immunofluorescence analysis on days 11 and 18. These specimens were preserved in 4% paraformaldehyde (PFA) and subsequently sectioned into slices of 5 µm thickness using a Cryostat. H&E staining was performed following standard procedures. For immunofluorescence histochemistry and immunohistochemistry, the primary antibodies employed included rabbit anti‐CD31 (Abcam, ab222783), rabbit anti‐CD34 (Abcam, ab81289), rabbit anti‐β‐catenin (Abcam, ab68183), rabbit anti‐Ki67 (Abcam, ab16667).

### RNA Sequencing

To unravel the mechanism of ROS‐dependent hair regrowth, the skins after treatment with Blank, PEA, PEA/NAC materials were collected on day 17. Total RNAs were extracted and the sequencing platform of illumina Novaseq6000 (Shanghai Biotechnology Corporation, China) was applied to obtain the gene expression profiles. The DEGs in the comparison of PEA versus PEA/NAc were introduced to KEGG pathway annotation and trend analysis.

### Statistical Analysis

Data preprocessing included outlier evaluation and normalization, with results expressed as mean ± SD. Two‐group comparisons performed a two‐tailed, unpaired Student’ s *t*‐test, while multi‐group comparisons applied a one‐way ANOVA with a Bonferroni post‐hoc test. Grouped data analysis involved a two‐way ANOVA. Statistical test assumptions were verified for validity. Above statistical evaluations were conducted using GraphPad Prism version 8 software (GraphPad Software Inc.). Statistical significance is set as *p < 0.05, **p < 0.01, ***p < 0.001, ****p < 0.0001; ns, not statistically significant.

## Conflict of Interest

The authors declare no conflict of interest.

## Supporting information



Supporting Information

## Data Availability

The data that support the findings of this study are available from the corresponding author upon reasonable request.

## References

[1] M. Ristow , Nat. Med. 2014, 20, 709. 24999941 10.1038/nm.3624

[2] G. A. Timblin , K. M. Tharp , B. Ford , J. M. Winchester , J. Wang , S. Zhu , R. I. Khan , S. K. Louie , A. T. Iavarone , J. Ten Hoeve , D. K. Nomura , A. Stahl , K. Saijo , Nat. Metab. 2021, 3, 618. 34031590 10.1038/s42255-021-00392-w PMC8162914

[3] J. Meng , Z. Lv , Y. Zhang , Y. Wang , X. Qiao , C. Sun , Y. Chen , M. Guo , W. Han , A. Ye , T. Xie , B. Chu , C. Shi , S. Yang , C. Chen , Antioxid. Redox Signaling 2021, 34, 1069. 10.1089/ars.2020.8212 PMC8080931 33270507

[4] J. Meng , Z. Lv , Y. Zhang , Y. Wang , X. Qiao , C. Sun , Y. Chen , M. Guo , W. Han , A. Ye , T. Xie , B. Chu , C. Shi , S. Yang , C. Chen , Antioxid. Redox Signaling 2020, 34, 1069. 10.1089/ars.2020.8212 PMC8080931 33270507

[5] H. Sies , Redox Biol. 2017, 11, 613. 28110218 10.1016/j.redox.2016.12.035 PMC5256672

[6] Y. Funato , T. Michiue , M. Asashima , H. Miki , Nat. Cell Biol. 2006, 8, 501. 16604061 10.1038/ncb1405

[7] R. B. Hamanaka , A. Glasauer , P. Hoover , S. Yang , H. Blatt , A. R. Mullen , S. Getsios , C. J. Gottardi , R. J. DeBerardinis , R. M. Lavker , N. S. Chandel , Sci. Signaling 2013, 6, ra8. 10.1126/scisignal.2003638 PMC4017376 23386745

[8] C. S. Gibhardt , S. Cappello , R. Bhardwaj , R. Schober , S. A. Kirsch , Z. Bonilla del Rio , S. Gahbauer , A. Bochicchio , M. Sumanska , C. Ickes , I. Stejerean‐Todoran , M. Mitkovski , D. Alansary , X. Zhang , A. Revazian , M. Fahrner , V. Lunz , I. Frischauf , T. Luo , D. Ezerina , J. Messens , V. V. Belousov , M. Hoth , R. A. Böckmann , M. A. Hediger , R. Schindl , I. Bogeski , Cell Rep. 2020, 33, 108292. 33086068 10.1016/j.celrep.2020.108292

[9] X. Zhang , M. D. Lee , C. Wilson , J. G. McCarron , Cell Calcium 2019, 84, 102108. 31715384 10.1016/j.ceca.2019.102108 PMC6891240

[10] D. Chen , Z. Yu , W. Wu , Y. Du , Q. Du , H. Huang , Y. Li , T. Xuan , Y.‐C. Liang , Y. Liu , Z. Wang , R. Su , Y. Zhao , Q. Li , M. Luo , F. Wang , J. Li , C.‐M. Chuong , Z. Lin , T. Chen , Cell 2025, 188, 5175. 40818454 10.1016/j.cell.2025.07.035 PMC13308630

[11] S. Noreng , N. Ota , Y. Sun , H. Ho , M. Johnson , C. P. Arthur , K. Schneider , I. Lehoux , C. W. Davies , K. Mortara , K. Wong , D. Seshasayee , M. Masureel , J. Payandeh , T. Yi , J. T. Koerber , Nat. Commun. 2022, 13, 6079. 36241643 10.1038/s41467-022-33711-0 PMC9568551

[12] L. A. Sena , N. S. Chandel , Mol. Cell 2012, 48, 158. 23102266 10.1016/j.molcel.2012.09.025 PMC3484374

[13] H. Sies , V. V. Belousov , N. S. Chandel , M. J. Davies , D. P. Jones , G. E. Mann , M. P. Murphy , M. Yamamoto , C. Winterbourn , Nat. Rev. Mol. Cell Biol. 2022, 23, 499. 35190722 10.1038/s41580-022-00456-z

[14] R. K. Tao , Y. Z. Zhao , H. Y. Chu , A. X. Wang , J. H. Zhu , X. J. Chen , Y. J. Zou , M. Shi , R. M. Liu , N. Su , J. L. Du , H. M. Zhou , L. Y. Zhu , X. H. Qian , H. Y. Liu , J. Loscalzo , Y. Yang , Nat. Methods 2017, 14, 720. 28581494 10.1038/nmeth.4306 PMC5555402

[15] Y. J. Zou , A. Wang , M. Shi , X. J. Chen , R. M. Liu , T. Li , C. X. Zhang , Z. Zhang , L. Y. Zhu , Z. Y. Ju , J. Loscalzo , Y. Yang , Y. Z. Zhao , Nat. Protoc. 2018, 13, 2362. 30258175 10.1038/s41596-018-0042-5 PMC6714056

[16] N. Klusch , M. Dreimann , J. Senkler , N. Rugen , W. Kühlbrandt , H.‐P. Braun , Nat. Plants 2023, 9, 142. 36585502 10.1038/s41477-022-01308-6 PMC9873573

[17] R. D. Guzy , B. Hoyos , E. Robin , H. Chen , L. Liu , K. D. Mansfield , M. C. Simon , U. Hammerling , P. T. Schumacker , Cell Metab. 2005, 1, 401. 16054089 10.1016/j.cmet.2005.05.001

[18] E. L. Bell , T. A. Klimova , J. Eisenbart , C. T. Moraes , M. P. Murphy , G. R. S. Budinger , N. S. Chandel , J. Cell Biol. 2007, 177, 1029. 17562787 10.1083/jcb.200609074 PMC2064363

[19] E. D. Getzoff , D. E. Cabelli , C. L. Fisher , H. E. Parge , M. S. Viezzoli , L. Banci , R. A. Hallewell , Nature 1992, 358, 347. 1353610 10.1038/358347a0

[20] C. L. Fisher , D. E. Cabelli , J. A. Tainer , R. A. Hallewell , E. D. Getzoff , Proteins 1994, 19, 24. 8066083 10.1002/prot.340190105

[21] J. Zhao , A. Blayney , X. Liu , L. Gandy , W. Jin , L. Yan , J.‐H. Ha , A. J. Canning , M. Connelly , C. Yang , X. Liu , Y. Xiao , M. S. Cosgrove , S. R. Solmaz , Y. Zhang , D. Ban , J. Chen , S. N. Loh , C. Wang , Nat. Commun. 2021, 12, 986. 33579943 10.1038/s41467-021-21258-5 PMC7881117

[22] M. Shen , Y. You , C. Xu , Z. Chen , BMC Complementary Med. Ther. 2024, 24, 147. 10.1186/s12906-024-04436-y PMC10996149 38580929

[23] A. E. Oro , Cell Stem Cell 2008, 2, 104. 18371427 10.1016/j.stem.2008.01.008 PMC3839661

[24] A. Gafter‐Gvili , B. Sredni , R. Gal , U. Gafter , Y. Kalechman , Am. J. Physiol. Cell Physiol. 2003, 284, C1593. 12734112 10.1152/ajpcell.00537.2002

[25] B. Zhang , T. Chen , Nat. Rev. Mol. Cell Biol. 2024, 25, 87. 37903969 10.1038/s41580-023-00662-3

[26] Y.‐C. Hsu , E. Fuchs , Cold Spring Harbor Perspect. Biol. 2022, 14, a040840. 10.1101/cshperspect.a040840 PMC8977401 34607830

[27] D. Huang , C. C. Zhang , M. Xiao , X. Li , W. C. Chen , Y. Jiang , Y. M. Yuan , Y. P. Zhang , Y. J. Zou , L. Deng , Y. Wang , Y. Y. Sun , W. P. Dong , Z. Zhang , L. Xie , Z. Yu , C. Q. Chen , L. G. Liu , J. Wang , Y. Yang , J. Yang , Y. Z. Zhao , J. K. Zheng , Proc. Natl. Acad. Sci. USA 2023, 120, 2210796120. 10.1073/pnas.2210796120 PMC10068762 36947513

[28] Y. Qu , X. Lu , Z. Xin , ACS Sustainable Chem. Eng. 2024, 12, 7739.

[29] T. Zhang , X.‐C. Zhong , Z.‐X. Feng , X.‐Y. Lin , C.‐Y. Chen , X.‐W. Wang , K. Guo , Y. Wang , J. Chen , Y.‐Z. Du , Z.‐M. Zhuang , Y. Wang , W.‐Q. Tan , Bioact. Mater. 2025, 45, 322. 39669127 10.1016/j.bioactmat.2024.11.028 PMC11635612

[30] Y. Zhong , F. Shahidi , J. Agric. Food Chem. 2011, 59, 6526. 21526762 10.1021/jf201050j

[31] X. Yang , S. H. M. Lim , J. Lin , J. Wu , H. Tang , F. Zhao , F. Liu , C. Sun , X. Shi , Y. Kuang , J. Y. H. Toy , K. Du , Y. Zhang , X. Wang , M. Sun , Z. Song , T. Wang , J. e. Wu , K. N. Houk , D. Huang , Nat. Commun. 2022, 13, 6424. 36307433 10.1038/s41467-022-34123-w PMC9614196

[32] L. Mignion , C. M. Desmet , E. Harkemanne , I. Tromme , N. Joudiou , M. Wehbi , J.‐F. Baurain , B. Gallez , Free Radical Biol. Med. 2022, 190, 226. 35987421 10.1016/j.freeradbiomed.2022.08.020

[33] H. Liu , X. Qu , E. Kim , M. Lei , K. Dai , X. Tan , M. Xu , J. Li , Y. Liu , X. Shi , P. Li , G. F. Payne , C. Liu , Biomaterials 2018, 162, 109. 29438879 10.1016/j.biomaterials.2017.12.027

[34] S. Grimaldi , R. Arias‐Cartin , P. Lanciano , S. Lyubenova , B. Endeward , T. F. Prisner , A. Magalon , B. Guigliarelli , J. Biol. Chem. 2010, 285, 179. 19892705 10.1074/jbc.M109.060251 PMC2804163

[35] M. C. Biesinger , L. W. M. Lau , A. R. Gerson , R. S. C. Smart , Appl. Surf. Sci. 2010, 257, 887.

[36] C. Kalha , L. E. Ratcliff , J. J. G. Moreno , S. Mohr , M. Mantsinen , N. K. Fernando , P. K. Thakur , T. L. Lee , H. H. Tseng , T. S. Nunney , J. M. Kahk , J. Lischner , A. Regoutz , Phys. Rev. B 2022, 105, 045129.

[37] N. Zhang , Y. Xiong , Adv. Sens. Energy Mater. 2023, 2, 100047.

[38] M. Wan , H. Chen , Q. Wang , Q. Niu , P. Xu , Y. Yu , T. Zhu , C. Mao , J. Shen , Nat. Commun. 2019, 10, 966. 30814497 10.1038/s41467-019-08670-8 PMC6393443

[39] Y. C. Sung , P. R. Jin , L. A. Chu , F. F. Hsu , M. R. Wang , C. C. Chang , S. J. Chiou , J. T. Qiu , D. Y. Gao , C. C. Lin , Y. S. Chen , Y. C. Hsu , J. Wang , F. N. Wang , P. L. Yu , A. S. Chiang , A. Y. Wu , J. J. Ko , C. P. Lai , T. T. Lu , Y. Chen , Nat. Nanotechnol. 2019, 14, 1160. 31740794 10.1038/s41565-019-0570-3

[40] Q. Pan , L. Xie , H. Zhu , Z. Zong , D. Wu , R. Liu , B. He , Y. Pu , Regener. Biomater. 2024, 11, rbae122. 10.1093/rb/rbae122 PMC11558062 39539979

[41] X. Zhao , Y. Liu , J. Am. Chem. Soc. 2021, 143, 9423. 34133170 10.1021/jacs.1c02186

[42] J. M. Mayer , J. Am. Chem. Soc. 2023, 145, 7050. 36943755 10.1021/jacs.2c10212 PMC10080693

[43] J. J. Warren , T. A. Tronic , J. M. Mayer , Chem. Rev. 2010, 110, 6961. 20925411 10.1021/cr100085k PMC3006073

[44] P. Li , Y. Jiao , Y. Ruan , H. Fei , Y. Men , C. Guo , Y. Wu , S. Chen , Nat. Commun. 2023, 14, 6936. 37907596 10.1038/s41467-023-42749-7 PMC10618200

[45] P. Kuleta , R. Pietras , J. Andrys‐Olek , A. Wójcik‐Augustyn , A. Osyczka , Phys. Chem. Chem. Phys. 2023, 25, 21935. 37551546 10.1039/d3cp02433d

[46] B. Tang , J. Zhao , J.‐F. Xu , X. Zhang , Chem. Sci. 2020, 11, 1192. 34123243 10.1039/c9sc06143f PMC8148027

[47] J. Zhao , C. Fu , K. Ye , Z. Liang , F. Jiang , S. Shen , X. Zhao , L. Ma , Z. Shadike , X. Wang , J. Zhang , K. Jiang , Nat. Commun. 2022, 13, 685. 35115516 10.1038/s41467-022-28346-0 PMC8813992

[48] Z. Sang , Y. Qiao , R. Chen , L. Yin , F. Hou , J. Liang , Nat. Commun. 2025, 16, 4050. 40307221 10.1038/s41467-025-58628-2 PMC12043898

[49] K. Jiang , S. Back , A. J. Akey , C. Xia , Y. Hu , W. Liang , D. Schaak , E. Stavitski , J. K. Nørskov , S. Siahrostami , H. Wang , Nat. Commun. 2019, 10, 3997. 31488826 10.1038/s41467-019-11992-2 PMC6728328

[50] G. Yang , J. Zhu , P. Yuan , Y. Hu , G. Qu , B.‐A. Lu , X. Xue , H. Yin , W. Cheng , J. Cheng , W. Xu , J. Li , J. Hu , S. Mu , J.‐N. Zhang , Nat. Commun. 2021, 12, 1734. 33741940 10.1038/s41467-021-21919-5 PMC7979714

[51] P. Rompolas , K. R. Mesa , V. Greco , Nature 2013, 502, 513. 24097351 10.1038/nature12602 PMC3895444

[52] J. J. Rennick , A. P. R. Johnston , R. G. Parton , Nat. Nanotechnol. 2021, 16, 266. 33712737 10.1038/s41565-021-00858-8

[53] L. von Kleist , W. Stahlschmidt , H. Bulut , K. Gromova , D. Puchkov , M. J. Robertson , K. A. MacGregor , N. Tomilin , A. Pechstein , N. Chau , M. Chircop , J. Sakoff , J. P. Kries , W. Saenger , H.‐G. Kräusslich , O. Shupliakov , P. J. Robinson , A. McCluskey , V. Haucke , Cell 2011, 146, 471. 21816279 10.1016/j.cell.2011.06.025

[54] C. J. Richards , T. C. Q. Burgers , R. Vlijm , W. H. Roos , C. Åberg , ACS Nano 2023, 17, 16517. 37642490 10.1021/acsnano.3c01124 PMC10510712

[55] L. Bai , Y. Wang , K. Wang , X. Chen , Y. Zhao , C. Liu , X. Qu , Adv. Mater. 2024, 36, 2311459. 10.1002/adma.202311459 38346345

[56] M. Hunt , M. Torres , E. Bachar‐Wikstrom , J. D. Wikstrom , Commun. Biol. 2024, 7, 1534. 39562800 10.1038/s42003-024-07219-w PMC11577046

[57] K. Khorsandi , R. Hosseinzadeh , H. Esfahani , K. Zandsalimi , F. K. Shahidi , H. Abrahamse , Inflammation Regener. 2022, 42, 40. 10.1186/s41232-022-00226-6 PMC9529607 36192814

[58] Y. Pan , S. Neuss , A. Leifert , M. Fischler , F. Wen , U. Simon , G. Schmid , W. Brandau , W. Jahnen‐Dechent , Small 2007, 3, 1941. 17963284 10.1002/smll.200700378

[59] S. M.‐Y. Fan , Y.‐T. Chang , C.‐L. Chen , W.‐H. Wang , M.‐K. Pan , W.‐P. Chen , W.‐Y. Huang , Z. Xu , H.‐E. Huang , T. Chen , M. V. Plikus , S.‐K. Chen , S.‐J. Lin , Proc. Natl. Acad. Sci. USA 2018, 115, E6880. 29959210 10.1073/pnas.1719548115 PMC6055137

[60] S. Müller‐Röver , K. Foitzik , R. Paus , B. Handjiski , C. van der Veen , S. Eichmüller , I. A. McKay , K. S. Stenn , J. Invest. Dermatol. 2001, 117, 3. 11442744 10.1046/j.0022-202x.2001.01377.x

[61] J. Ma , C. Qin , J. Wu , H. Zhuang , L. Du , J. Xu , C. Wu , Mater. Horiz. 2023, 10, 3773. 37409407 10.1039/d3mh00528c

[62] K. N. Li , P. Jain , C. H. He , F. C. Eun , S. Kang , T. Tumbar , eLife 2019, 8, 45977. 10.7554/eLife.45977 PMC6684267 31343406

[63] Y. Xiao , W.‐M. Woo , K. Nagao , W. Li , A. Terunuma , Y.‐s. Mukouyama , A. E. Oro , J. C. Vogel , I. Brownell , J. Invest. Dermatol. 2013, 133, 2324. 23558405 10.1038/jid.2013.167 PMC3742722

[64] R. Sennett , M. Rendl , Semin. Cell Dev. Biol. 2012, 23, 917. 22960356 10.1016/j.semcdb.2012.08.011 PMC3496047

[65] Y.‐C. Hsu , L. Li , E. Fuchs , Cell 2014, 157, 935. 24813615 10.1016/j.cell.2014.02.057 PMC4041217

[66] Y.‐C. Hsu , H. A. Pasolli , E. Fuchs , Cell 2011, 144, 92. 21215372 10.1016/j.cell.2010.11.049 PMC3050564

[67] Y. V. Zhang , J. Cheong , N. Ciapurin , D. J. McDermitt , T. Tumbar , Cell Stem Cell 2009, 5, 267. 19664980 10.1016/j.stem.2009.06.004 PMC2756832

[68] J. Liu , Q. Xiao , J. Xiao , C. Niu , Y. Li , X. Zhang , Z. Zhou , G. Shu , G. Yin , Signal Transduction Targeted Ther. 2022, 7, 3. 10.1038/s41392-021-00762-6 PMC8724284 34980884

[69] V. Horsley , A. O. Aliprantis , L. Polak , L. H. Glimcher , E. Fuchs , Cell 2008, 132, 299. 18243104 10.1016/j.cell.2007.11.047 PMC2546702

[70] J. Ding , S.‐J. Lee , L. Vlahos , K. Yuki , C. C. Rada , V. van Unen , M. Vuppalapaty , H. Chen , A. Sura , A. K. McCormick , M. Tomaske , S. Alwahabi , H. Nguyen , W. Nowatzke , L. Kim , L. Kelly , D. Vollrath , A. Califano , W.‐C. Yeh , Y. Li , C. J. Kuo , Nat. Commun. 2023, 14, 2947. 37268690 10.1038/s41467-023-37689-1 PMC10238527

[71] K. Lai , I. Pritišanac , Z.‐Q. Liu , H.‐W. Liu , L.‐N. Gong , M.‐X. Li , J.‐F. Lu , X. Qi , T.‐L. Xu , J. Forman‐Kay , H.‐B. Shi , L.‐Y. Wang , S.‐K. Yin , Nature 2024, 631, 826. 38987597 10.1038/s41586-024-07684-7 PMC11269185

[72] C. Qin , S. Yang , Y.‐H. Chu , H. Zhang , X.‐W. Pang , L. Chen , L.‐Q. Zhou , M. Chen , D.‐S. Tian , W. Wang , Signal Transduction Targeted Ther. 2022, 7, 215. 10.1038/s41392-022-01064-1 PMC9259607 35794095

[73] S. A. Lipton , Nat. Rev. Drug Discovery 2006, 5, 160. 16424917 10.1038/nrd1958

[74] G. F. Clunn , P. S. Sever , A. D. Hughes , Int. J. Cardiol. 2010, 139, 2. 19523699 10.1016/j.ijcard.2009.05.019 PMC2824626

[75] G. R. Crabtree , E. N. Olson , Cell 2002, 109, S67. 11983154 10.1016/s0092-8674(02)00699-2

[76] P. G. Hogan , Cell Calcium 2017, 63, 66. 28153342 10.1016/j.ceca.2017.01.014 PMC5739523

[77] T. Lu , F. Chen , J. Comput. Chem. 2012, 33, 580. 22162017 10.1002/jcc.22885

