# Supplementary material for: Nitrogen‐Boosted H2O2 Production of Arginine‐Polyphenol Nanozyme Drives Oxidative Eustress for Hair Regeneration
Source: Adv Sci (Weinh). 2025 Nov 14;13(18):e19561. doi: 10.1002/advs.202519561 (PMC13042590; doi:10.1002/advs.202519561)

**Supporting Information for**

**Nitrogen-Boosted H_2_O_2_ Production of Arginine-Polyphenol Nanozyme Drives Oxidative Eustress for Hair Regeneration**

Yifei Wang ^a 1^, Yaojia Yang ^a 1^, Aoxue Wang ^b,c,d^, Chen Shen ^b^, Shenliang Tan ^a^, Changsheng Liu ^a^, Yuzheng Zhao ^b,c,d^ *, and Xue Qu ^a,c^ *

*^a^ Key Laboratory for Ultrafine Materials of Ministry of Education, Engineering Research Center for Biomedical Materials of Ministry of Education, Frontiers Science Center for Materiobiology and Dynamic Chemistry, School of Materials Science and Engineering, East China University of Science and Technology, Shanghai, 200237, China*

*^b^ Optogenetics & Synthetic Biology Interdisciplinary Research Center, State Key Laboratory of Bioreactor Engineering, School of Pharmacy, East China University of Science and Technology, Shanghai 200237, China*

*^c^ Shanghai Frontiers Science Center of Optogenetic Techniques for Cell Metabolism, East China University of Science and Technology, Shanghai, 200237, China*

*^d^ Research Unit of New Techniques for Live-cell Metabolic Imaging, Chinese Academy of Medical Sciences, Beijing 100730, China*

* Corresponding authors.

*E-mail addresses:*

[quxue@ecust.edu.cn](mailto:quxue@ecust.edu.cn)

yuzhengzhao@ecust.edu.cn

^1^The two authors contributed equally to this work.


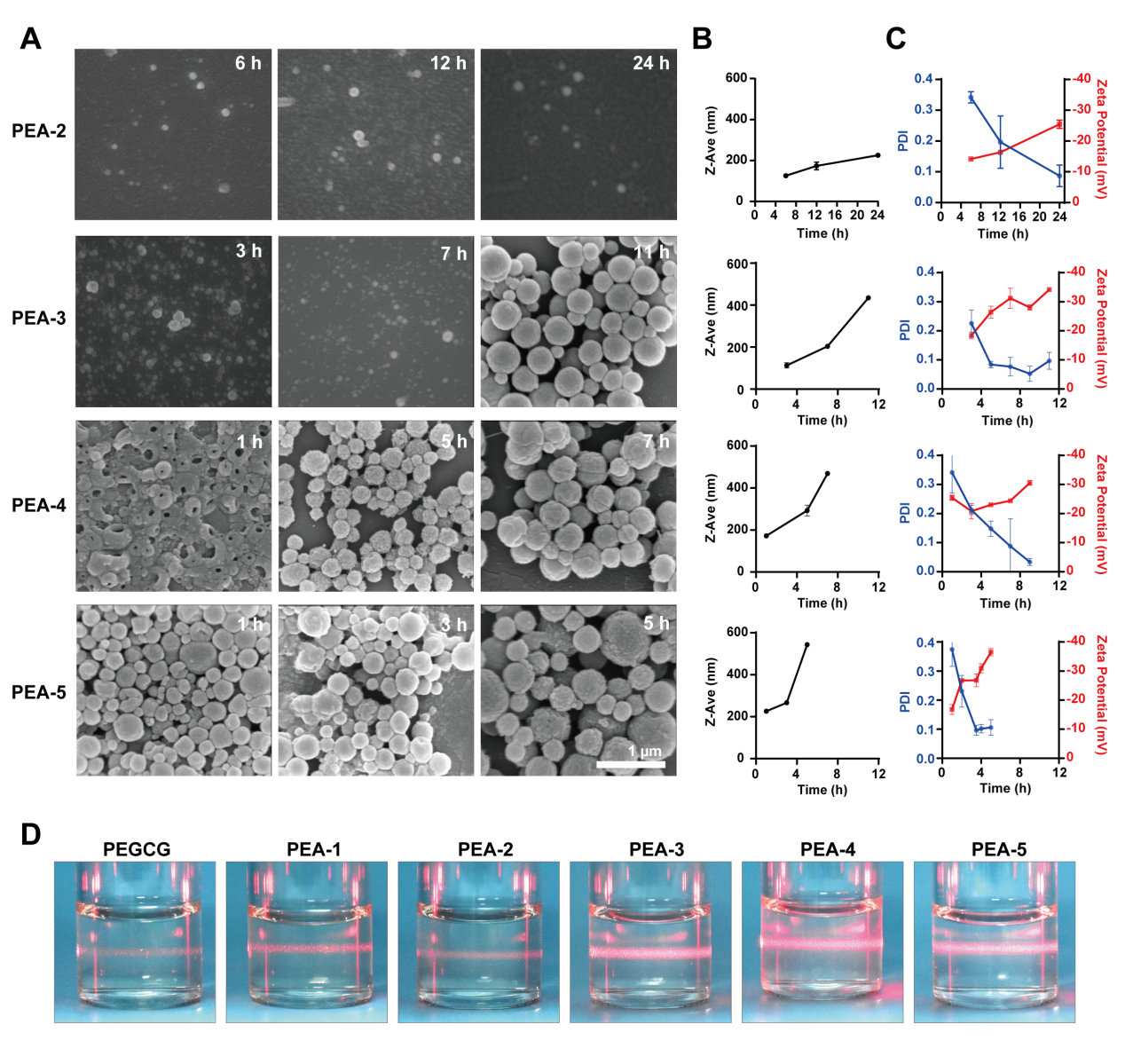


**Figure S1.** (A) SEM morphology images of different PEAs during the synthesis process. (B) Z-averaged hydrodynamic size change curves of different PEAs during the synthesis process (n=3). (C) PDI and zeta potential change curves of different PEAs during the synthesis process (n=3). (D) PEAs suspended in PBS and their tyndall effects.


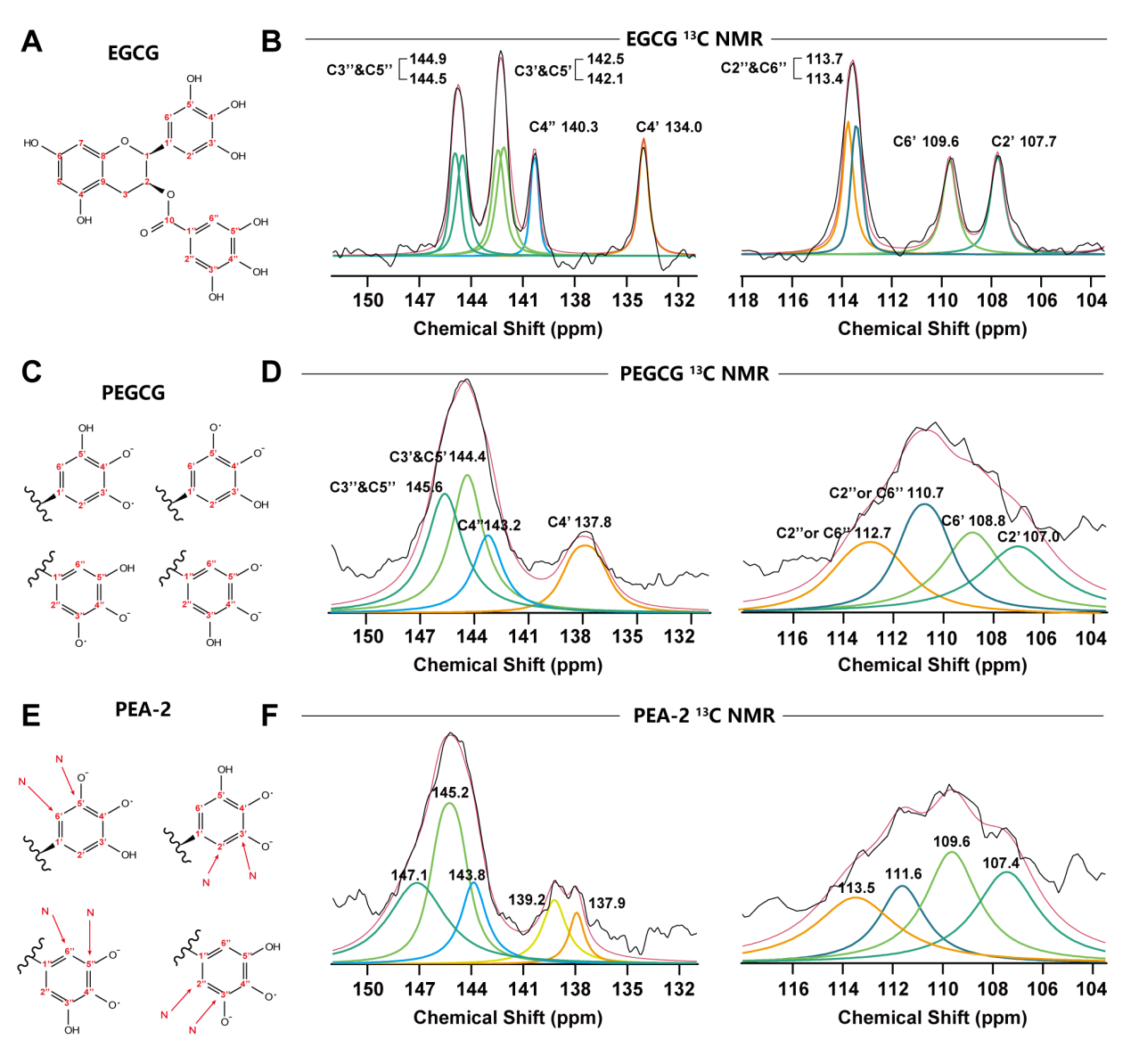


**Figure S2.** (A) Molecular structural formula and (B) ¹³C NMR spectrum of EGCG. (C) Possible characteristic structural formula of PEGCG after oxidative polymerization and its (D) ¹³C NMR spectrum. (E) Possible structural formula of PEA-2 obtained through polymerization mediated by L-Arg and its (F) ¹³C NMR spectrum.


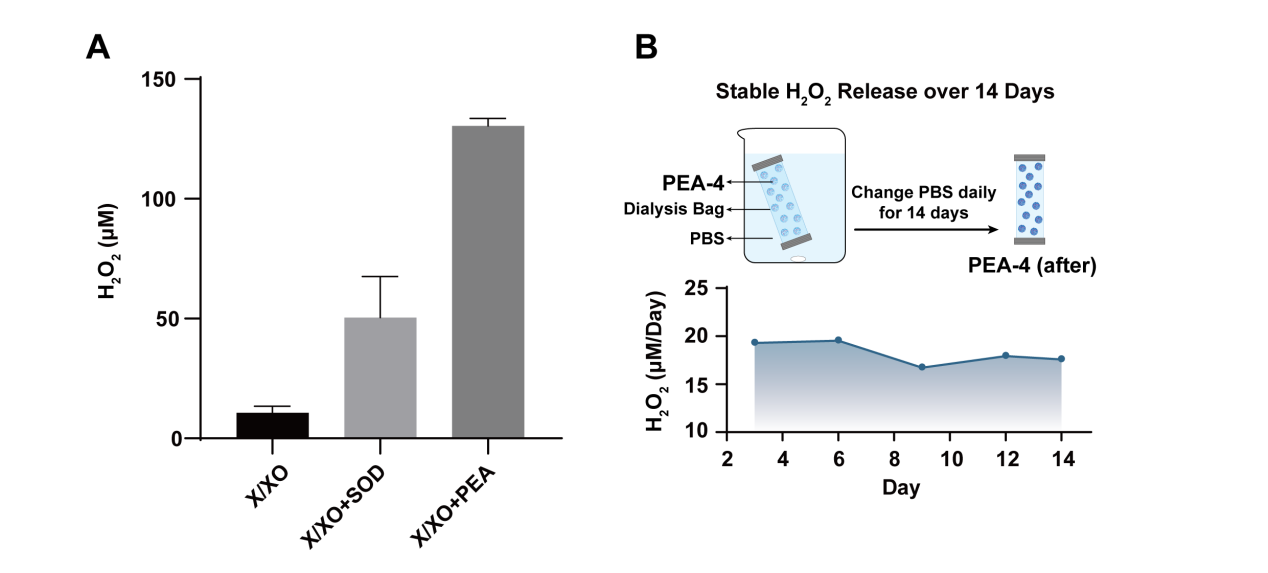


**Figure S3.** (A) H_2_O_2_ release after addition of SOD enzyme and PEA-4 to the xanthine/xanthine oxidase system (n=3). (B) H_2_O_2_ release stability assessment of PEA-4 following 14 days of continuous operation.


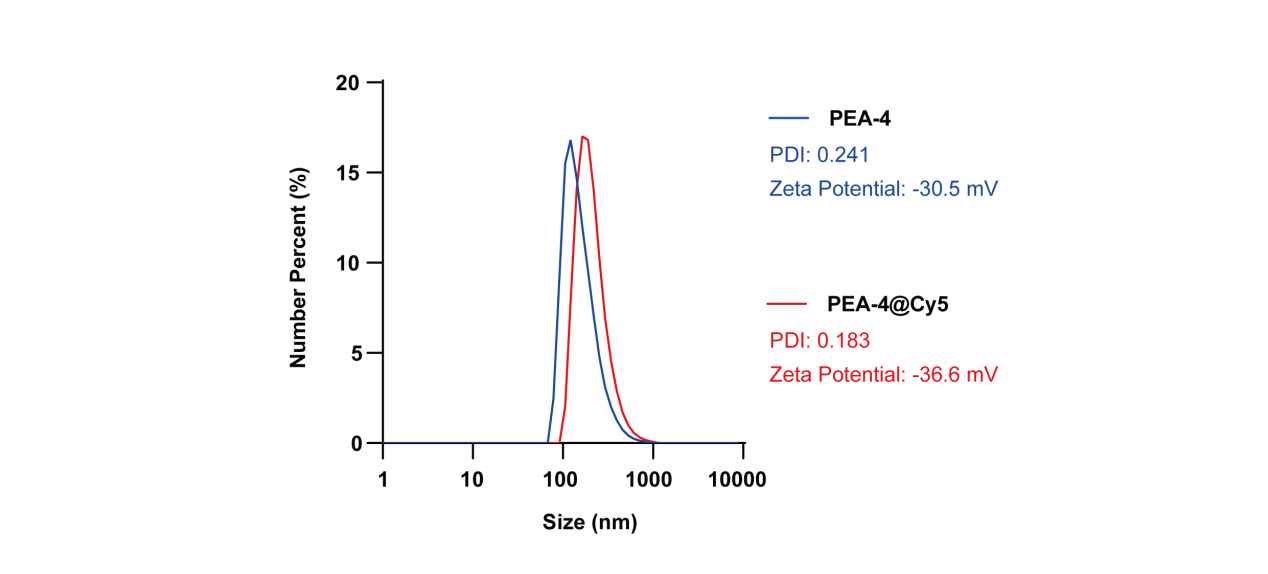


**Figure S4.** Hydration kinetic size and zeta potential before and after Cy5-NH_2_ modification.


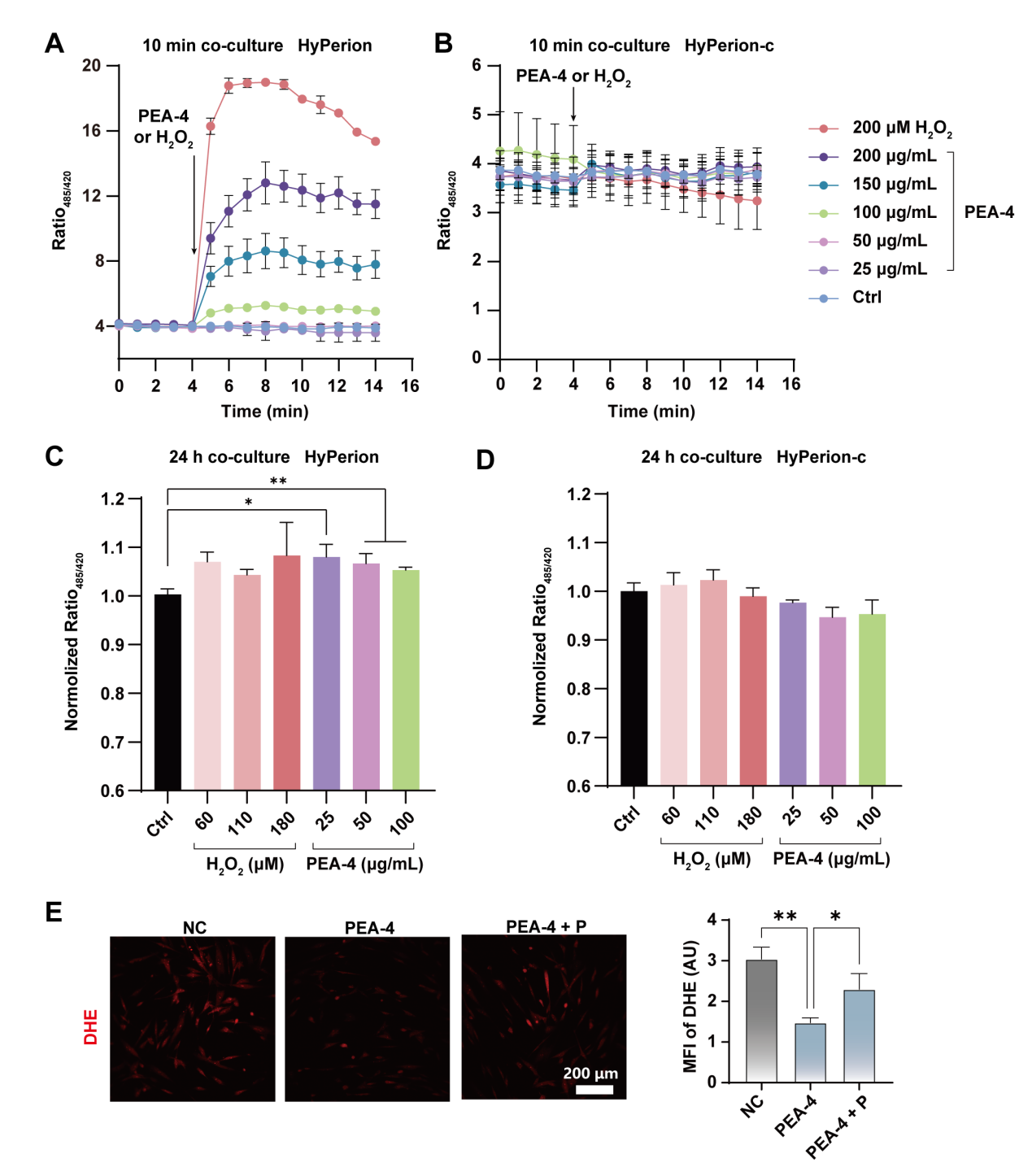


**Figure S5.** (A) Kinetics of the HyPerion fluorescence response in HeLa cells upon different concentrations of PEA-4 or H_2_O_2_ addition (n=3). (B) Kinetics of the HyPerion-c fluorescence response in HeLa cells upon different concentrations of PEA-4 or H_2_O_2_ addition within 10 min, HyPerion-c displays as a pH corrector (n=3). (C) Fluorescence ratio detection of HeLa cells expressing HyPerion treated with different concentration of PEA-4 or H_2_O_2_ for 24 h. Data were normalized to the fluorescence of the control (n=3). (D) Fluorescence ratio detection of HeLa cells expressing HyPerion-c treated with different concentration of PEA-4 or H_2_O_2_ for 24 h, HyPerion-c displays as a pH corrector. Data were normalized to the fluorescence of the control (n=3). (E) PEA-4, possessing SOD-like enzyme activity, reduces intracellular superoxide anion levels, an effect inhibited by Pitstop 2 (n=3). Data were presented as the mean ± SD, and p values were obtained by unpaired two-tailed Student’s t tests; ns, p > 0.05, *p < 0.05, **p < 0.01 and ***p < 0.001.


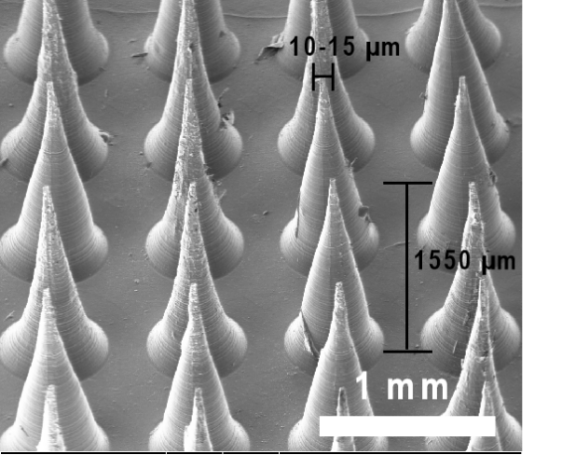


**Figure S6.** SEM micrograph of PEA-4 MNs, with the image annotations indicating the needle tip width and needle length, respectively.

**Table S1.** Synthesis formula for PEAs, mass percentage of N and O elements


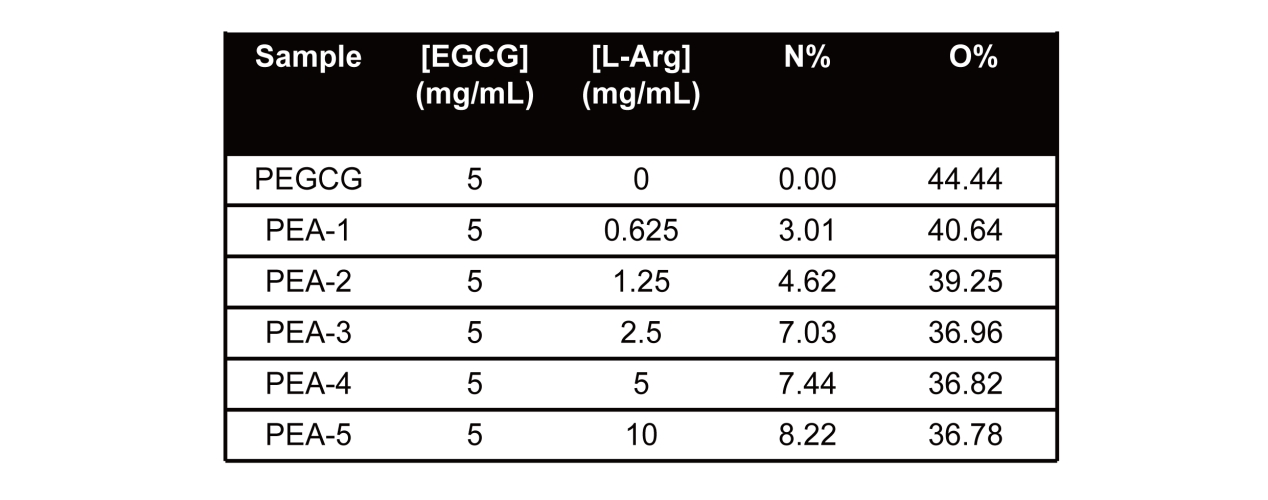

Supplement: Supplementary file 1 — Supporting Information [file ADVS-13-e19561-s001.docx]
